# Sow serenity: automatic long-term measurement of lying behavior in crates and free-farrowing pens using 3D accelerometers

**DOI:** 10.1093/jas/skae101

**Published:** 2024-04-06

**Authors:** Maximilian Knoll, Lorenz Gygax, Edna Hillmann

**Affiliations:** Humboldt-Universität zu Berlin, Department of Life Sciences, Albrecht Daniel Thaer Institute of Agricultural and Horticultural Sciences, Animal Husbandry and Ethology, 10099 Berlin, Germany; Humboldt-Universität zu Berlin, Department of Life Sciences, Albrecht Daniel Thaer Institute of Agricultural and Horticultural Sciences, Animal Husbandry and Ethology, 10099 Berlin, Germany; Humboldt-Universität zu Berlin, Department of Life Sciences, Albrecht Daniel Thaer Institute of Agricultural and Horticultural Sciences, Animal Husbandry and Ethology, 10099 Berlin, Germany

**Keywords:** activity, animal welfare, free farrowing, pig, sensor

## Abstract

Accelerometers are useful in analyzing lying behavior in farm animals. The effect of the farrowing system on sow lying behavior has been studied around parturition, but not long-term. In a natural environment, sows increase activity 14 d post parturition, which we expected to be also evident in housed sows when they can move freely. The objective of this study was (1) to validate the methodology to automatically measure sow lying bouts and duration with accelerometers and (2) to apply it to crated and free-farrowing sows 24-h pre-parturition until weaning. We used videos with manual behavior coding as the gold standard for validation and calculated the agreement with an intraclass correlation coefficient (ICC), which was 0.30 (95% CI: −0.10 to 0.64) for the number of lying bouts. When transitional sitting bouts were excluded from the video dataset, the ICC for lying bouts increased to 0.86 (95% CI: 0.40 to 0.95). For lying duration, the ICC was 0.93 (95% CI: 0.26 to 0.98). We evaluated the effects of housing, day relative to parturition, and time of day on lying using the accelerometer data and linear mixed models. In crated sows, the number of lying bouts increased toward parturition, peaking at about five bouts per 6 h, and decreased to almost zero bouts after parturition. Then, it increased again (*P* = 0.001). In free-farrowing sows, the number of lying bouts gradually decreased from a high level towards parturition and was lowest after parturition. It remained constant, as in the crated sows, until day 15, when the number of bouts increased to eight bouts on day 20 (*P* = 0.001). Sows in both systems were lying almost all of the time between 18:00 and 00:00 hours and on all days (*P* = 0.001). The crated sows showed a very similar pattern in the other three-quarters of the day with a reduced lying time before parturition, a peak after parturition, reduced lying time from days 5 to 20, and an increase again towards weaning (*P* = 0.001). Free-farrowing sows had a similar pattern to the crated sows from 00:00 to 06:00 hours, but without the reduction in lying time from days 5 to 20. They showed an increase in lying time toward parturition, which remained constant with a final decrease toward weaning, especially during the day (*P* = 0.001). This study proves the accuracy of accelerometer-based sow lying behavior classification and shows that free-farrowing systems benefit lactating sows around parturition but also towards weaning in the nest-leaving phase by facilitating activity.

## Teaser

This study demonstrates the feasibility of automatically measuring the lying behavior of sows using triaxial accelerometers. Long-term application from the day pre-parturition throughout the entire lactation period in two farrowing environments demonstrated the need for sows to move freely during the period associated with nest building and nest abandonment.

## Introduction

Sows spend a majority of their time lying, and in intensive housing systems will lie for approximately 70% to 80% of the day (Buckner et al., 1998; Bergeron et al., 2000). Lying behavior is particularly important around parturition and during lactation as it varies according to the specific biological needs of the sow and her litter. Baxter et al. (2011) identify three phases with distinct behavioral characteristics: (1) nest building, (2) parturition, early lactation, and nest occupation, and (3) nest departure, with higher activity in phases 1 and 3 compared to phase 2, where lying is predominant. Sows also have a strong motivation to engage in behaviors such as nesting, foraging, socializing, and exploring when they are not lying (Dellmeier and Friend, 1991). Notably, these behavioral predispositions have persisted throughout the domestication process, with modern sows’ expression of these behaviors being influenced by their housing conditions (Broom et al., 1995; Baxter et al., 2018). Thus, sow lying behavior, and inversely, their activity level, may be an indicator of welfare reflecting their ability to satisfy physical and behavioral needs for rest or movement in various housing systems, especially during parturition and lactation.

Farrowing crates, which are still commonly used to prevent piglet crushing, hinder sows from meeting their physical and behavioral needs during farrowing. In the initial nest-building phase, sows exhibit increased activity 12 to 24 h before farrowing regardless of their housing system, illustrating the behavioral necessity of this process to the pre-parturient sow (reviewed by Wischner et al. [2009]). In a natural environment, post-parturition sows during early lactation primarily engage in nursing and caring for piglets, often lying down for prolonged periods (up to 90% of the time) to facilitate suckling and leaving the nest only for elimination behavior (Gundlach, 1968; Jensen, 1986). About 14 d post parturition, sows will leave the nest to return to their group, a phase characterized by increased foraging and feed intake, hence reflecting the natural desire for sows to increase their activity at this time (Jensen and Redbo, 1987). In common farrowing crate systems, pregnant sows are placed in farrowing crates about 5 d before they are due to give birth, and they remain in the crates until the piglets are weaned at about 28 d of age. The restricted movement and lack of access to nesting materials during the nest-building phase results in physiological distress (Lawrence et al., 1994). The crates prevent the sows not only from engaging in nest-building activity but also disrupt the biological needs of the sow to eliminate in places located away from the litter during the phase of early lactation, and from being able to increase foraging activity during the nest departure phase.

Several studies have investigated the differences in sow activity between farrowing crates and alternative farrowing systems without fixation (Andersen et al., 2014; Chidgey et al., 2016; Bolhuis et al., 2018; Loftus et al., 2020). However, these studies primarily focus on the first two phases a few days before and after parturition through direct or video observations. However, as reviewed in detail by Baxter et al. (2011), 7 to 14 d postpartum sows and piglets become more active due to the nest-leaving and social integration phase (Gundlach, 1968). In a natural environment, sows increase foraging behavior and feed intake during this period of increased metabolic demand for lactation (Baxter et al., 2011). Therefore, we expect that this increase in activity should also be seen in housed sows when they are allowed to move freely. The primary objective of this study was therefore to continuously measure and compare lying bouts and duration of lying from the day pre-parturition throughout the entire lactation period of sows in farrowing crates and free-farrowing pens under commercial conditions. We hypothesized that sows in both systems would show increased activity prior to farrowing due to nest-building, but that free-farrowing sows would have a higher level of pre-farrowing activity than crated sows. We also expected increased lying times post-parturition during early lactation for both sets of sows, but in general free-farrowing sows would have higher activity levels in late lactation than crated sows.

However, continuously measuring sow activity, especially over a period longer than a few days, is challenging, and traditional methods such as direct observation or video recording are time-consuming, labor-intensive, and prone to human error or bias. Therefore, there is a need for reliable and valid methods to assess sow activity in different housing conditions that are feasible and cost-effective.

An established method to automatically record lying is the use of triaxial accelerometers, which measure the acceleration of an object along three axes (x, y, and z). Accelerometers can be attached to the sow’s neck with collars, to the ears with tags, or to the back or legs with adhesive, and record movements continuously and automatically (reviewed by Chapa et al. [2020]). Several studies have already demonstrated the feasibility of using accelerometers to detect postures in sows (reviewed by Chapa et al. [2020]). Accelerometers have been used in a variety of contexts, such as monitoring the lying behavior and activity levels of periparturient sows housed in farrowing crates with the goal to monitor health disorders around farrowing and behavior of sows at risk of piglet crushing (Cornou et al., 2011); automatic onset of farrowing in sows (Traulsen et al., 2018); and determination of the beginning and end of nest building behavior to determine the ideal time for limited fixation at the time of farrowing (Oczak et al., 2015, 2019).

While all studies describe the methodology and mathematical formulas used to extract the data of interest from the raw accelerometer data, the underlying algorithms and analysis code are for the most part not freely available. This means that the scientific principle of repeatability is not fully granted due to a lack of information about the calculations performed and a lack of standardization. One notable exception is a recently validated study detailing the use of accelerometers to measure the number and duration of lying bouts in dairy cows, for which the underlying physics and algorithms have also recently been described in detail and implemented in the open-source R package ‘triact’ (Brouwers et al., 2023; Simmler and Brouwers, 2024).

Thus, the secondary objective of this study was to adopt a comprehensible approach to data collection and analysis in lactating sows, and thereby validate the method of using 3D accelerometers to detect lying, originally developed for cows by Simmler and Brouwers (2024), as a tool to achieve our primary objective.

## Materials and Methods

### Ethical considerations

This research was conducted in accordance with the German Animal Welfare Act (TierSchG), the German Animal Welfare Ordinance (TierSchNutztV), and the German Ordinance on Animal Experiments (TierSchVersV). The responsible authority (State Office for Occupational Safety, Consumer Protection, and Health, Brandenburg, Germany; Reference V6-2347/0-2022-5) has approved this study. All animals in the study were monitored daily and no adverse effects were observed from the measurement devices used.

### Farm, Housing, and Animals

**Figure 1.**
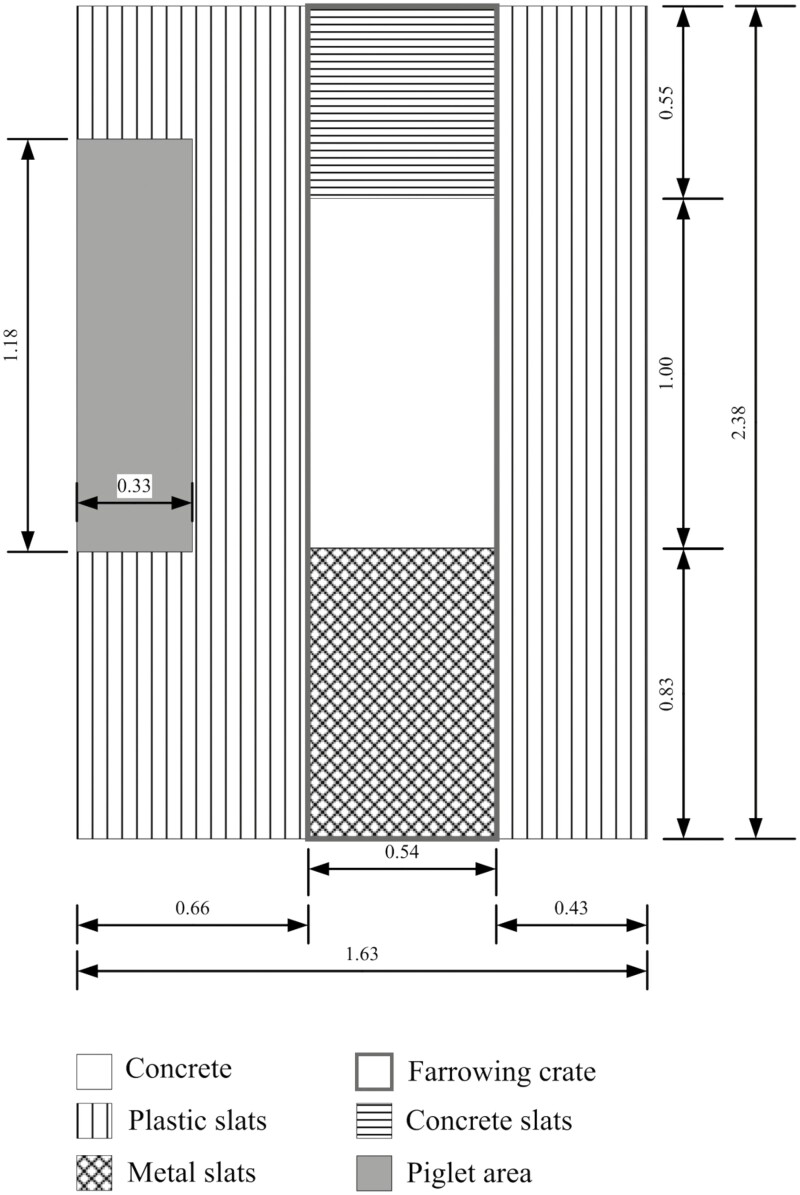
Layout of the pens with farrowing crates used in the trial.

We conducted our research on a commercial pig farm in the German state of Brandenburg in May and November 2022. Within this farm, we used six pens with farrowing crates and six free-farrowing pens. The barn containing the farrowing crates was an uninsulated building with forced ventilation. All farrowing crate pens had the same dimensions with a total area of 3.88 m^2^. The farrowing crate itself was 0.54 m wide, 2.0 m long, and 110 cm high. The flooring of the farrowing crates consisted of metal slats at the back (0.45 m^2^, 12% of the total area, 1 cm slat width), a solid floor in the center (0.54 m^2^, 14%), and a partially slatted concrete floor under the raised feed trough at the front (0.3 m^2^, 8%, Figure 1). The piglet areas were 0.4 m^2^ (10%) in size and consisted of solid fireclay, equipped with an infrared lamp. The remainder of the pen (2.2 m^2^, 57%) consisted of plastic slats with a slat width of 0.5 cm.

Crated sows were fed liquid diets consisting of wheat, barley, soybean extraction meal, wheat gluten, cereal bran, sugar beet pulp, calcium carbonate, soybean oil, sunflower extraction meal, cattle salt, palm oil, and monocalcium phosphate (crude protein: 17%; crude fat: 3.9%; crude fiber: 5.1%; and crude ash: 5.7%) eight times per day. Farrowing crate sows and piglets had constant access to water via a bowl drinker.

No bedding was provided for farrowing crate sows, but a jute sack was provided as nesting material 24 h prior to parturition.

**Figure 2.**
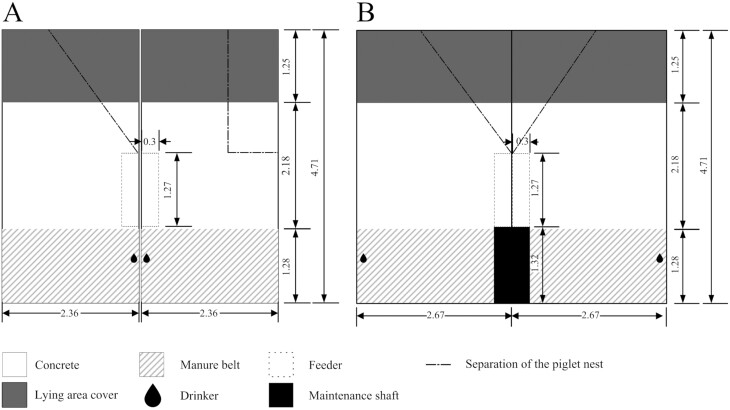
Layout of the free-farrowing pens used in the trial without (A) and with (B) maintenance shaft. In (A), the separation of the piglet nest is also shown in the position in which it could be used to fixate the sow (right pen).

The free-farrowing sows were housed in an uninsulated barn with natural ventilation (roof vents and side inlets), with four pens measuring 10.73 m^2^ and two measuring 12.58 m^2^ (with a usable area of 11.05 m²). The latter two had a maintenance shaft for the manure belt included in the pen (Figure 2). The manure belt provided an elimination area at the front of each pen and served as a “pig toilet” (3.01 m^2^, 28/27% of the pen area; PigT, Big Dutchman AG, Vechta, Germany). This elimination area was constructed of a perforated movable belt that allowed urine to drain away while removing any solid manure components and remaining organic material from the pen through rotation into a collection channel. The remainder of the pen was a solid concrete floor (7.73 m^2^, 72/70%), of which 4.78 m^2^ (45/43%) was an activity area with a feeder and a nipple drinker, and 2.95 m^2^ (27%) was a lying area covered with a lid (Figure 2). The pen layout was originally designed for wean-to-finish management (Havito, Big Dutchman AG, Vechta, Germany; not marketed for farrowing) and all other pens in the barn, except those used in the trial, housed weaners only. As the farm wanted to test the possibility of a birth-to-finish concept, the pens were retrofitted with a partition made of metal pipes allowing a separate piglet nest under the lid of the lying area, which could also be used to temporarily fixate the sows, for instance as a safety measure when vaccinating the piglets. After the first batch of piglets in May, bars 20 cm above ground were additionally installed on the walls opposite the feeders to provide support for the sows when lying down to prevent piglet crushing. Infrared heaters were not installed, but floor heating was provided throughout the area under the lid (Figure 2). Wood shavings and maize silage were provided daily by the farm staff as bedding and foraging material. No additional nest-building material was provided prior to parturition. The barn’s automatic feeding system dispensed feed for the weaners in the pens, not housing sows, but it was not technically feasible to convert only

certain pens on the automatic feeding system to sow feed. Therefore, dry feed identical in composition to the farrowing crate feed was manually fed to the sows in the trough twice daily. Three nipple drinkers per pen, located at the partition to the adjacent pen, provided water ad libitum to the free-farrowing sows (Figure 2).

In total, we observed 24 Danish Landrace sows, 12 housed in farrowing crates and 12 in free-farrowing pens for this study. In both systems, we used first parity gilts (“naive”) and multiparity sows with prior experience in farrowing crates (Table 1).

**Table 1.** Distribution of first parity gilts and multiparous sows between the housing systems in the two seasons

	Farrowing crate		Free farrowing	
Number of parturitions	May	November	May	November
0 to 1	1	0	0	6
2 to 8	5	6	6	0

### Data collection

#### Video recordings

**Figure 3.**
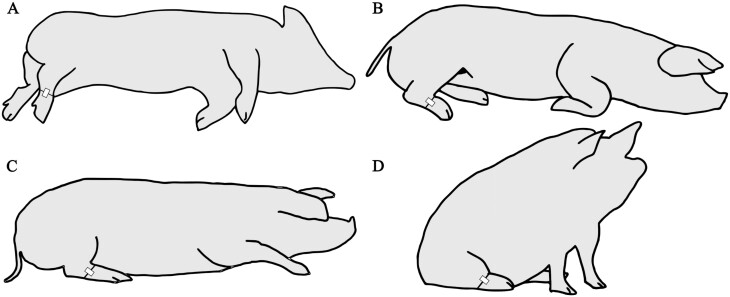
Leg and accelerometer positions of sows in natural lying (A: lateral, B: sternal, C: ventral) and D: sitting positions.

We equipped all pens with digital cameras (Dahua HFW2431S-S-S2, Dahua Technology Co., Ltd, Hangzhou, China) in the spring period. For validation, we chose the recordings from the second and 15th d after parturition, as they exemplified phase 2 (parturition) and phase 3 (nest leaving) with expected potential differences in lying behavior, and thus together were more representative of the general sample. Two observers each continuously analyzed a 24-h period (a total of 12 pigs × 2 d = 24 d) and recorded the start and end times of bouts of lying using the BORIS software (version 7.13.8, Friard and Gamba, 2016). Each observer completed observations in both systems, so that each observer evaluated 1 d with six sows in the crates and six sows in the free-farrowing system. We defined a bout as continuous period during which lying behavior was exhibited without interruption with lying defined as all instances in which the body was not supported by the legs, touching the ground ventrally or laterally, possibly with the legs tucked under the body (Figure 3).

#### Accelerometers

**Figure 4.**
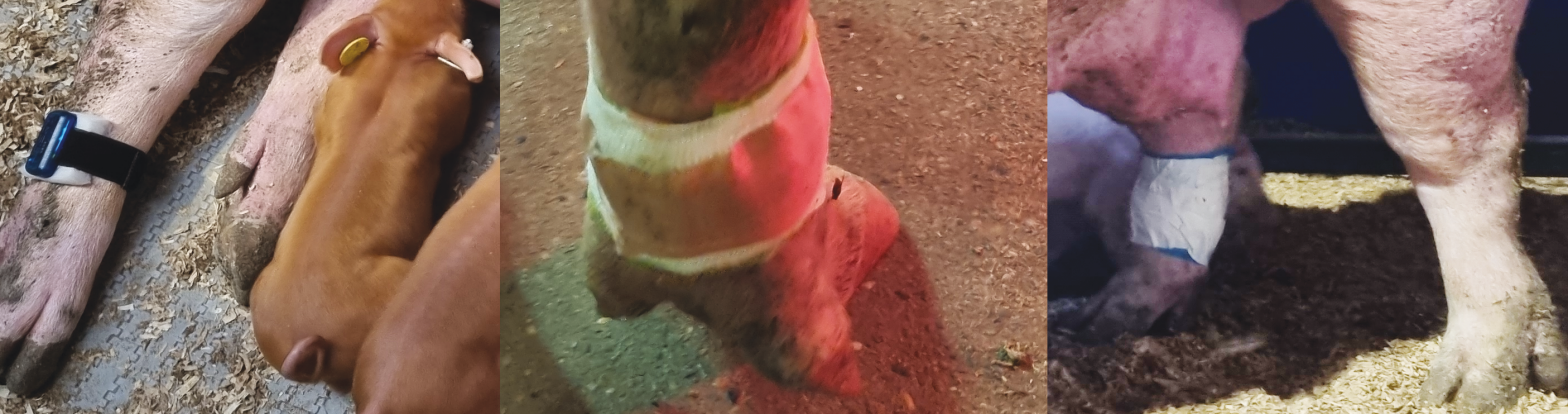
Accelerometer attached to the sows’ hind leg with Velcro and foam padding (left, first layer), gauze (middle, second layer), and tape (right, third layer).

To automatically record the number and duration of lying bouts, we fitted all sows with 3D accelerometers (approximately 20 g; MSR145B data logger, MSR Electronics GmbH, Seuzach, Switzerland, 27 × 16 × 53 mm). Before starting the measurements, we calibrated the accelerometers according to the manufacturer’s instructions by orienting all three axes vertically in both directions (MSR Electronics GmbH, 2016). We attached the loggers to one hind leg with a Velcro strap. We padded the loggers with foam to prevent skin abrasion. We also protected them with self-adhesive binding and tape. The protection was to prevent moisture penetration and manipulation by the piglets moving freely in the pen after birth (Figure 4).

We attached the accelerometers 24 h before expected parturition, which was induced with prostaglandin on day 115 of gestation, and removed them 1 d before weaning. We will refer to the period including the days before and after parturition as days relative to parturition. We replaced the loggers every 7 d when internal memory was close to full. The spring dataset included a total of 24 d, but one sow farrowed 4 d after the expected parturition date. In the fall dataset, most animals had a total of 26 d of data, but one sow farrowed 2 d before, two sows farrowed 1 d before, and two sows farrowed 1 d after the expected parturition date.

We programmed the loggers to record acceleration values at 1 Hz and used the y-axis, which is the axis parallel to the leg, to automatically detect lying. As described in the study by Simmler and Brouwers (2024), the y-axis of a standing sow measures approximately 1 g of gravitational acceleration because the leg is more or less perpendicular to the ground surface. When lying down, the tarsus is more or less parallel to the ground surface, resulting in a y-axis acceleration value close to zero. The leg position in the sitting position is similar to that in the lying position (Figure 3).

#### Data Preparation

For validation, we had to exclude parts of two sows’ records because the accelerometer was changed in the morning of 1 d, and because one accelerometer had a faulty recording schedule that resulted in a full record earlier than planned. We had to exclude one sow’s data for one of the selected validation days due to a faulty accelerometer recording, resulting in 21 full and two partial days for validation, a total of 23 d out of a planned 24.

We used the R-package ‘triact’ to determine the number of lying bouts and the lying duration from the raw accelerometer data (Simmler and Brouwers, 2024). We set the critical lying value to 0.75, so that we assigned each data point to either lying or standing based on whether the acceleration was greater or less than 0.75 *g*, respectively. We chose this relatively restrictive value because the sows did not put their legs down completely flat. To account for variability caused by movement, we set the window size to 32, in which a moving median was applied.

For validation, we selected the raw accelerometer data from the same days (24 h) as recorded and analyzed by video. To compare the farrowing systems, we segmented the data from each day into four 6-h periods (morning, afternoon, early night, and late night). The day of parturition was considered to be day 0 relative to parturition.

### Statistical analysis

For statistical calculations, we used the software R (version 4.3.1, R Core Team, 2023).

#### Validation

**Figure 5.**
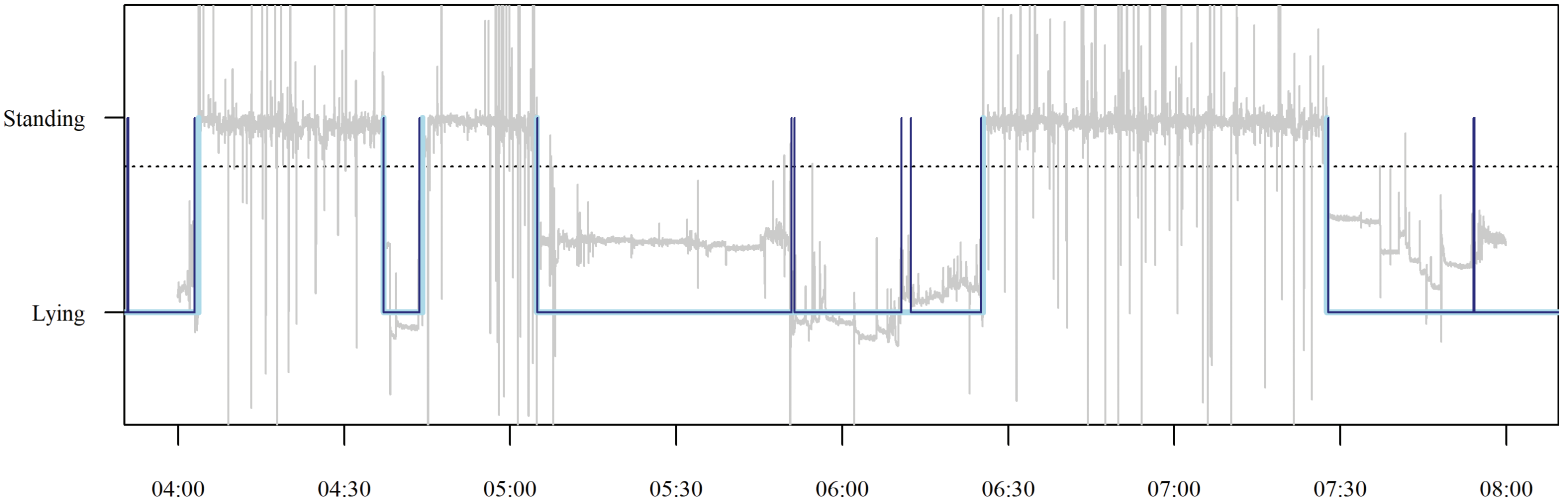
Raw accelerometer data (gray lines), *triact* lying classification (light bluelines), and video analysis lying classification (dark blue lines) for 4 h. The dotted line represents the critical lying value of 0.75 *g*. From about 5:05 to 6:20 hours, in the accelerometer data a single lying bout is classified, but in the video data lying is interrupted by two sitting bouts.

To validate the methodological approach of using accelerometers to automatically measure lying bouts using the ‘triact’ package, we first visually superimposed the lying time curves calculated from the accelerometer data and the video analysis (Figure 5, Supplementary Materials).

This showed that, for video analysis, we had marked the end of a lying bout when the animal was sitting. However, the accelerometers only detected when a pig rose to a fully standing posture, resulting in a lower total number of lying bouts (cf. the orientation of the accelerometer in the different postures, Figure 3). We manually counted the ‘sitting bouts’ detected in the videos in the superimposed curves and removed them from the number of lying bouts in the video dataset.

From the video and the accelerometer datasets, we calculated the total duration of lying and the number of lying bouts per 24-h period. We then compared these two aspects for both methods using an intraclass correlation coefficient (ICC) with a two-way random-effect model on 23 subjects (representing sows and 24-h periods) specified in the ‘icc’ command of the ‘irr’ R package (Gamer et al., 2012). We report the mean ICC estimates along with their 95% confidence intervals (CI).

#### Effect of housing system

We evaluated the long-term accelerometer data using Bayesian Linear Mixed-Effects Models (BLMER) with the function ‘blmer’ of the ‘blme’ package (Chung et al., 2013). We used the log-transformed number of lying bouts and the logit-transformed proportion of the lying duration in each quarter of a day as the outcome variables of a statistical model. The log- and logit-transformations were needed to ensure normality of the residuals in the models. Fixed effects included the housing system (farrowing crate or free farrowing), days relative to parturition, time of day (4-level factor: morning, afternoon, early night, and late night), and all interactions. Days relative to parturition were normalized and included in the models as a natural spline. We determined a potentially optimal degree of freedom for the spline using a generalized additive mixed models model based on the ‘gamm4’ package (Wood and Scheipl, 2020) including main effects only. This approach suggested 9 °C of freedom for the spline in the lying bout and 6 °C of freedom in the lying duration model. We compared this choice for our BLMER models with models reduced and increased by 3 °C of freedom (i.e., 6 and 12 for the lying bouts and 3 and 9 for lying duration) until there was no statistical support for further improvement.

Animal identification was nested in batches (spring and fall) as a random effect. The number of previous parturitions (Table 1) partially accounted for the variation between animals. As parturition was not fully balanced with batch and housing system, we did not include it as a fixed effect per se (see Discussion). The identity of each 6-h period was included as a crossed random effect to account for the simultaneous measurement of sows.

We visually assessed the homogeneity of variance assumptions of the models using a Tukey-Anscombe plot and the normality of the residuals using the ‘simulateResiduals’ function (package ‘DHARMa’; Hartig, 2022) and of random effects using Q-Q plots. Finally, we visually assessed the residuals for equal distribution by plotting the residual against the fixed effects. We did not observe major deviations from the assumptions in either model after the applied transformations.

We used sum contrasts for both categorical fixed effects. The use of sum contrasts when comparing reduced models with the full model provides interpretable main effects, even in the presence of interactions (Schad et al., 2020). First, we compared the full model, including all fixed effects, to a null model, including only the intercept. To obtain individual *P*-values for fixed effects or interactions, we compared models reduced by the effect or interaction of interest with the full model. For model comparisons, we used a parametric bootstrap as implemented in the ‘PBmodcomp’ function from the ‘pbkrtest’ package (Halekoh and Højsgaard, 2014). We obtained model estimates and corresponding CI through 1.000 parametric bootstrap simulations using the ‘bootMer’ function in ‘lme4’ (Bates et al., 2015).

## Results

### Validation

**Figure 6.**
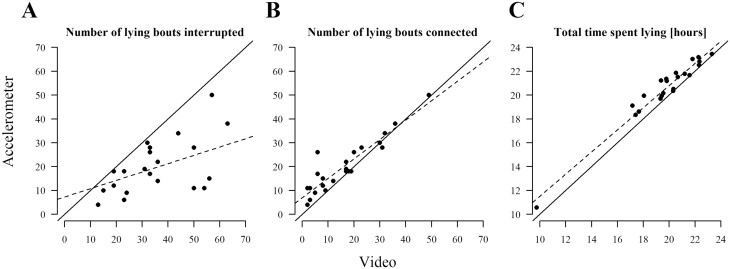
(A) and (B) show the number of lying bouts as evaluated by the accelerometer vs. based on video data. In (A) sitting bouts as recorded in the videos were taken to interrupt lying and initiate a new lying bout, in (B) sitting bouts were taken to connect the same lying (resting) bout. (C) shows the total time spent lying over 24 h. The solid line indicates a 1:1 relationship. The dashed line is a simple regression for illustration.

The ICC, or the agreement between the accelerometer and video data, was 0.30 (95% CI: −0.10 to 0.64) for the original number of lying bouts (Figure 6 A). When sitting bouts between lying bouts, before standing up, or before lying down were excluded from the video dataset, the ICC for lying bouts increased to 0.86 (95% CI: 0.40 to 0.95, Figure 6 B). For lying duration, the ICC was 0.93 (95% CI: 0.26 to 0.98, Figure 6 C) without correction for sitting.

### Effect of housing system

Our models with 9 °C of freedom for modeling the time course with a natural spline fit better than models with only 6 °C of freedom (number of lying bouts: *P* = 0.02; lying duration: *P* = 0.05). There was no statistical support for further improvement with 12 °C of freedom compared to only 9 °C of freedom (number of lying bouts: *P* = 0.39; lying duration: *P* = 0.57).

**Figure 7.**
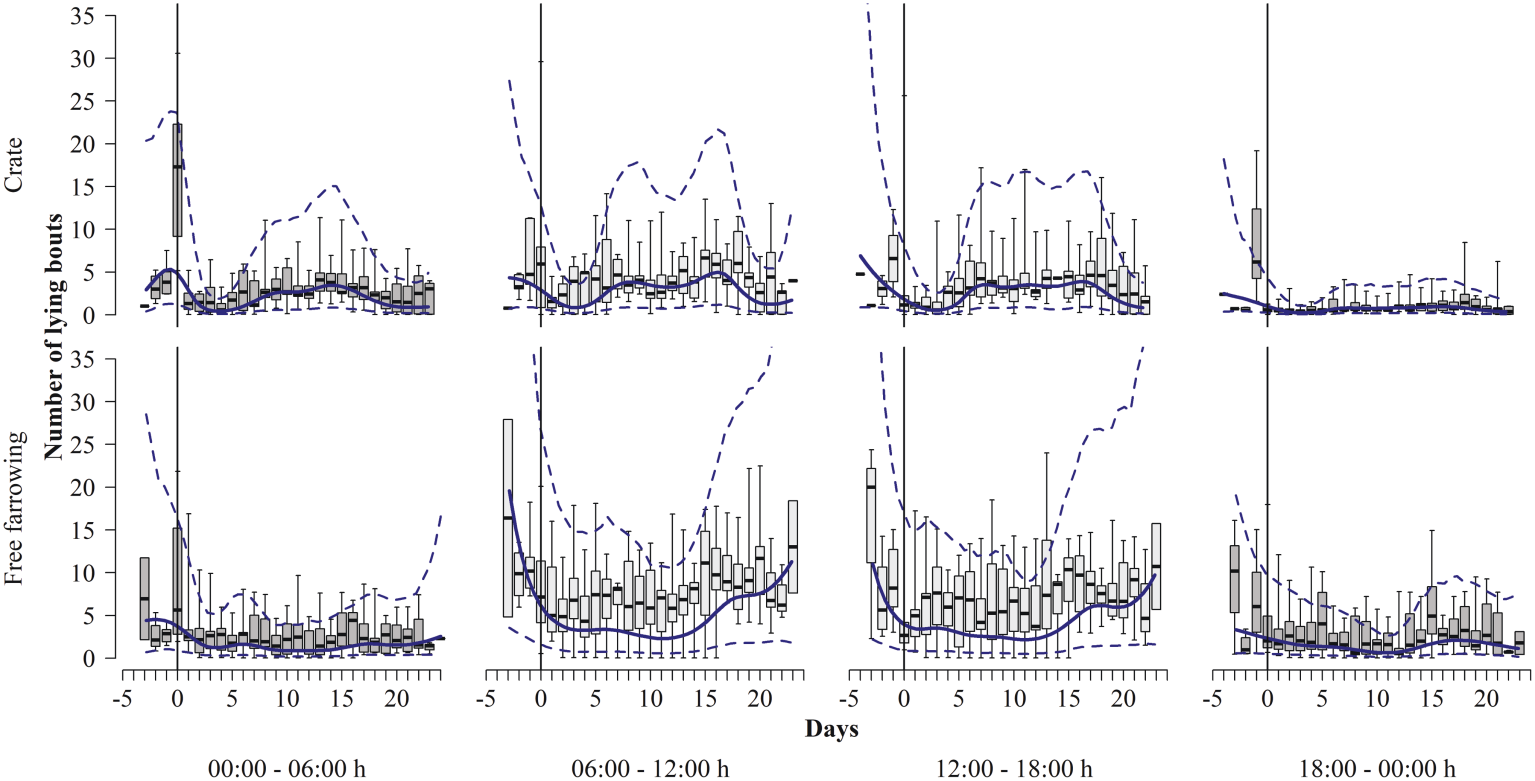
Frequency of lying bouts observed in both crated and free-farrowing sows relative to the day of parturition, marked by a solid vertical line at day 0. Data are further stratified by the time of day, with dark boxplots representing raw nighttime data and light gray boxplots representing daytime observations. Bold lines represent model estimates and dashed lines represent 95% confidence intervals.

In crated sows, the number of lying bouts increased toward parturition, where it peaked at about five bouts per 6 h and decreased to almost zero bouts immediately after the day of parturition. Thereafter, the number of lying bouts increased again to a moderate number (Table 2, Figure 7).

**Table 2.** Global *P*-value and *P*-values for main effects and interactions for the outcome variables number of lying bouts and lying duration

	Number of lying bouts	Lying duration
Global	0.001	0.001
*Main effects*		
System	0.12	0.07
Day relative to parturition	0.001	0.001
Time of day	0.18	0.004
*Two-way interactions*		
System × day relative to parturition	0.001	0.001
System × time of day	0.85	0.62
Day relative to parturition × time of day	0.44	0.001
*Three-way interaction*		
System × day relative to parturition × time of day	0.92	0.001

In free-farrowing sows, the number of lying bouts gradually decreased from a high level on day −3 towards the day of parturition and was lowest on the days after parturition. It remained constant, as in the crated sows, until day 15, when the number of bouts increased to eight bouts on day 20 (Table 2, Figure 7).

**Figure 8.**
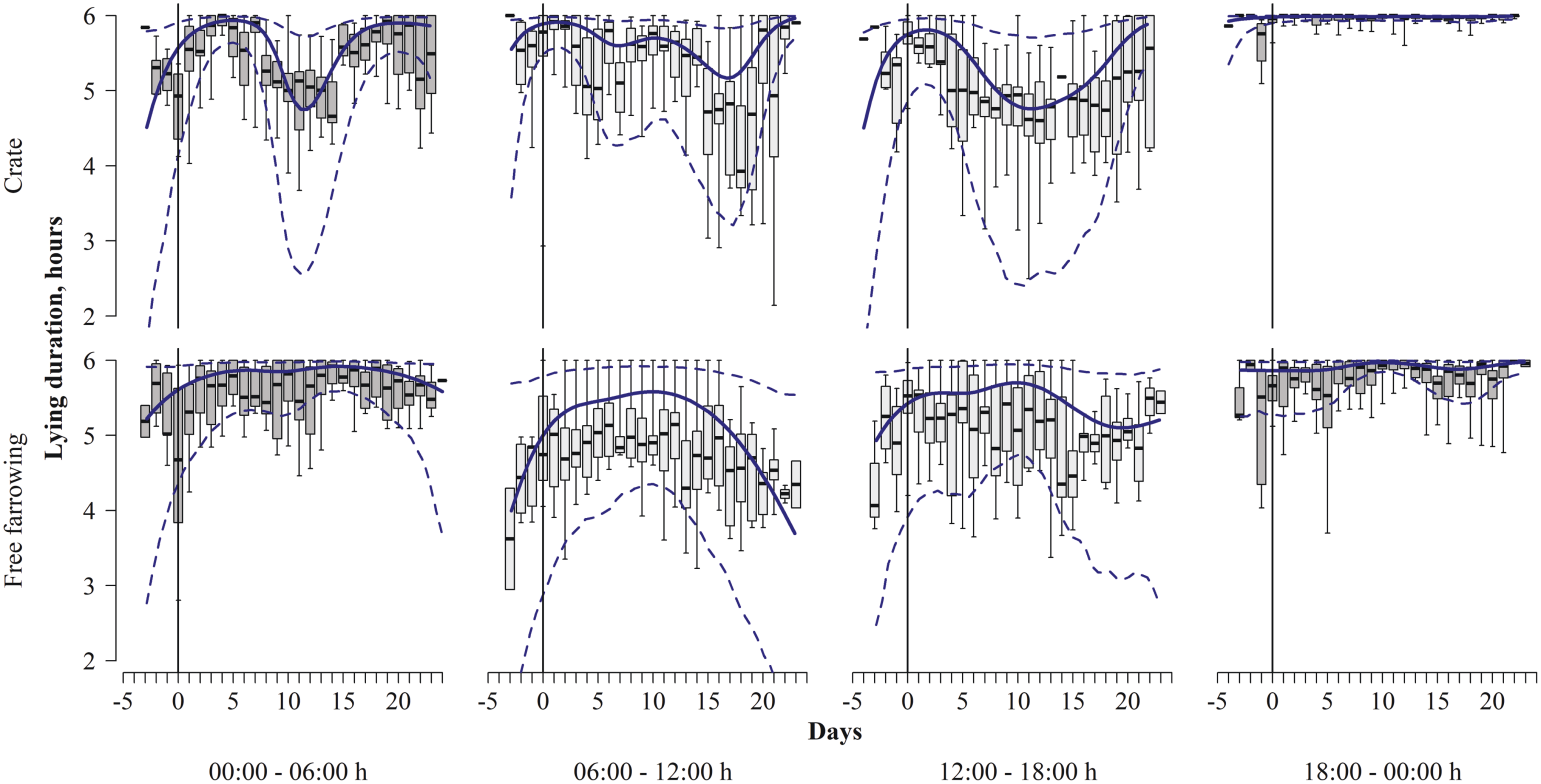
Lying duration was observed in both crated and free-farrowing sows relative to the day of parturition, marked by a solid vertical line at day 0. Data are further stratified by the time of day, with dark boxplots representing raw nighttime data and light gray boxplots representing daytime observations. Bold lines represent model estimates and dashed lines represent 95% confidence intervals.

Sows in both systems were lying almost all of the time between 18:00 and 00:00 hours and on all days (Table 2, Figure 8).

The crated sows showed a very similar pattern in the other three-quarters of the day with a reduced lying time before parturition, a peak after parturition, reduced lying time from days 5 to 20, and an increase again towards weaning (Table 2, Figure 8).

Free-farrowing sows had a similar pattern to the crated sows from 00:00 to 06:00 hours, but without the reduction in lying time from days 5 to 20. They showed an increase in lying time toward parturition, which remained constant with a final decrease toward weaning, especially during the day (Table 2, Figure 8).

## Discussion

### Validation

Our approach for automatically detecting the onset and duration of lying in sows using accelerometers attached to the sow’s leg and data processing via the ‘triact’ package has proven to be reliable and robust. It is worth noting, however, that unlike cows, sows often exhibit sitting behavior after getting up or before lying down. In the video analysis, sitting bouts were used to interrupt lying and to initiate a new lying bout, but since the position of a sow’s legs is the same whether she is lying or sitting (cf. Figure 3), the algorithm could not distinguish between these two postures when analyzing accelerometer data. However, since the algorithm correctly determined the duration of lying, even though it could not use sitting bouts to interrupt lying and initiate a new lying bout, it is reasonable to assume that the time sows spend in the sitting position is relatively short and serves as a transitional posture. With this in mind, we argue that it is tolerable to neglect sitting bouts for most research questions.

### General methodology

Compared to previous work, the approach used in this study was simple because it only distinguished between a lying and a standing position. Depending on how accelerometers are attached to the animals and what calculations are performed, it is generally also possible to distinguish whether a sow is lying laterally (Cornou and Lundbye-Christensen, 2008, 2010; Ringgenberg et al., 2010; Escalante et al., 2013) and on which side (Thompson et al., 2016, 2019), ventrally (Cornou and Lundbye-Christensen, 2008; Ringgenberg et al., 2010) or sternally (Cornou and Lundbye-Christensen, 2010; Cornou et al., 2011; Escalante et al., 2013; Thompson et al., 2016, 2019). In addition, accelerometers can distinguish between standing and walking (Thompson et al., 2019), detect sitting (Ringgenberg et al., 2010; Thompson et al., 2019), depict postural transitions (Thompson et al., 2016, 2019), and identify rooting behavior (Cornou and Lundbye-Christensen, 2008, 2010; Escalante et al., 2013).

However, while these studies found it possible to discriminate between different lying positions using accelerometers, they could not satisfactorily discriminate between standing and sternal lying positions when accelerometers were placed on sow’s backs or necks (Thompson et al., 2019). In some cases, discrimination was not attempted at all and was instead classified as medium active behavior, corresponding to standing, sitting, or lying (Cornou et al., 2011). Classifying sitting behavior has proven to be particularly difficult as Ringgenberg et al. (2010) correctly predicted sitting in only 37% of all cases, Thompson et al. (2019) could not robustly discriminate between sternal recumbency and sitting, and Cornou et al. (2011) combined sitting, standing and sternal lying into “medium activity”. These authors also followed our line of argument that sitting is largely a transitional posture. However, the ability to differentiate between sitting and transitional positions is important in obtaining more data on behavioral risk factors for crushing events, as discussed by Thompson et al. (2016), and as supported by data from Vieuille et al. (2003) where 27% of crushing events occurred in a sitting position. In general, there appear to be no differences in the amount of time sows spend sitting when comparing crates and free-farrowing systems (Blackshaw et al., 1994; Chidgey et al., 2016; Loftus et al., 2020).

With this in mind, and depending on the research question, it may be beneficial to add an additional sensor on the neck or back to record more nuanced behaviors while keeping the accelerometer on the leg, which would then allow for accurate prediction of standing as shown in this study. In general, it may also be possible to attach an additional accelerometer to the front leg of the sow. However, there is a high risk of damage to the sensor as the animal could easily reach and manipulate it. Especially in the days around parturition, it might be of interest to measure the exact lying position of a sow, as only a lateral position gives the piglets access to the udder and could therefore be an indicator of good maternal behavior. Additionally, lying down from a standing position, and rolling from a ventral or sternal to a lateral position are the greatest postural transition risks for piglet crushing (Weary et al., 1998), so studying these behaviors requires accurate discrimination between standing and sternal lying. In principle, the ‘triact’ package has implemented the ability to use the axes of multiple sensors to perform these calculations using a leg and a neck sensor (Brouwers et al., 2023; Simmler and Brouwers, 2024).

Nevertheless, a simple, easy-to-implement and reproducible approach, such as the one described in this study, is often sufficient to provide evidence of underlying health and welfare problems (Weary et al., 2009; Matthews et al., 2016, 2017).

### Effect of housing system and possible confounding effects

#### Farrowing system

The higher number of observed lying bouts in sows combined with decreased lying duration in the days before parturition confirmed our hypothesis and is well in line with the nest-building behavior described in the literature (reviewed by Wischner et al. [2009]). The fact that the number of lying bouts was higher and lying duration was lower in free-farrowing sows compared to crated sows suggests that sows with the ability to move freely are more able to satisfy the intrinsic need to perform nest-building behavior and will do so when given the opportunity.

Previous studies have used accelerometers to determine optimal time points for temporary confinement in the days before parturition, i.e., when nest-building behavior is completed and the onset of farrowing is detected (Oczak et al., 2015, 2019). In our long-term measurement, we found additional dynamics that give reason to critically assess the crating of sows for the entire lactation period: The decreased lying time observed in the free-farrowing sows at about 10 d postpartum could be an expression of the nest-leaving behavior reviewed by Baxter et al. (2011). Although the sows were not able to leave the farrowing pen or to reunite with the other sows as they would do in a natural environment, they may have been motivated to be active after early lactation.

In our study, the farrowing systems were partly confounded with other variables that are discussed below (4.3.3 ff).

#### Piglet loss rate

The average piglet loss rate for live-born piglets in the trial was 17% for the crated and 41% for the free-farrowing sows, which is much higher than reported for established free-farrowing systems (Weber et al., 2007). However, there is evidence that free-farrowing alone is not necessarily the sole cause of piglet losses. Factors such as litter size at birth, sow age, season at birth, and organization of pen structures into separate nesting and activity areas all play an important role in piglet loss (Weber et al., 2007; Wechsler and Weber, 2007). Breeding and management must work together in free-farrowing systems to minimize the risk factors for crushing events. The number of piglets per litter should be in a range that ensures good birth weight and high viability. Sows should exhibit good maternal behavior and the design of the farrowing pen must allow for such, i.e., they should gather the piglets in a safe place before lying down, and they should be allowed appropriate space to lie down carefully to prevent crushing piglets (Weber et al., 2009).

The free-farrowing system used in this study did not fit the above-described recommendations to ensure piglet safety in free-farrowing housing environments and may have contributed to the observed higher rates of piglet loss by crushing. The farm staff was not trained prior to the trial, and had no experience caring for sows in a free-farrowing environment. The highly productive breed used had an average of 17 live-born piglets per litter in both farrowing systems during the trial. Additionally, under the existing farm management, litters were balanced by moving individual piglets from a sow with a high litter size to sows with lower litter sizes. The breed and management were therefore probably not well suited to free farrow housing, a**s** there were too many piglets for the sow to appropriately manage without constraint. Although the free-farrowing pens were an appropriate size to house the sows, the pens were not structured adequately to provide a separate nest and activity area. Appropriate temperature regulation posed a further challenge to sow and piglet welfare. Since the majority of the pens in the free-farrowing barn housed only weaners, the heat in the barn was adjusted to meet the thermal needs of young pigs and not sows. As a result, the barn temperature was likely too high for the sows, and we frequently observed them lying in the elimination area, presumably to cool down. It is also possible that the floor heating did not provide enough warmth to the piglets. The addition of a heat lamp and suitable nesting material would have rectified this issue as well as further structuring the nest area.

#### Nest-building material

Appropriate nesting materials for pigs have the characteristics of being complex, changeable, moveable, and manipulable (Wischner et al., 2009). As discussed by Bolhuis et al. (2018), the jute sack provided in the farrowing crates and the wood shavings in the free-farrowing pens were most likely not suitable for nesting. It is possible that sows in both systems would have spent less time lying if they had been provided with adequate nesting material on the day before parturition. For instance, Baxter et al. (2011) recommend providing per sow at least 2 kg of long-stemmed straw prepartum. As the long-term measurements in this study showed a decrease in lying behavior in free-farrowing sows towards weaning, it would be interesting to investigate whether the provision of adequate nesting material also influences lying behavior over longer periods of time. This may be particularly relevant until the onset of the nest-leaving phase, as evidenced by Gundlach’s (1968) observations that wild boars consistently engage in nest-building activities whenever they return to the original nest site. Wild boars will scrape and collect scattered materials and return them to the edges of the nest up to 14 d after parturition.

#### Parturition status and previous system experience

To eliminate the influence on behavior of prior experience in a free-farrowing environment, none of the animals in the trial had previously been housed in free-farrowing pens. However, after the high number of piglet losses in May, the farm decided to use only gilts in the free-farrowing system in November so as not to jeopardize the offspring of valuable breeding animals. This resulted in an unbalanced distribution of parturition status across batch and housing systems. However, parturition status influences sow behavior and the risk of piglet loss. Gilts lack experience in litter care, and piglet mortality decreases from first to second parity (King et al., 2019). Older, multiparous sows tend to have larger litters and piglets with lower birth weights (Weary et al., 1998), both of which are risk factors for piglet crushing (Weber et al., 2009). Despite these possible influences, there was no difference in piglet mortality in the free-farrowing system between the two batches. It can be argued that multiparous sows are more experienced in litter care, which may have partially offset the risks of piglet crushing by older sows.

Research by King et al. (2019) also showed that sows which were housed in different systems from one farrow to the next had higher piglet mortality rates. This suggests that exposure to restrictive environments during one farrowing period would negatively affect subsequent farrowing behavior in less confined, free-farrowing systems, as noted by King et al. (2018). This highlights the importance of a consistent farrowing environment in influencing maternal behavior and piglet survival from one farrow to the next. In our study, the inexperience of the gilts in litter care and the prior exposure of the multiparous sows to crate housing probably influenced piglet crushing.

## Conclusions

Given the overall excellent agreement between the accelerometer measurements and video analysis as gold standard, we consider the described methodology to be a valuable analytical tool with a high degree of repeatability. The long-term application showed that free-farrowing systems benefit lactating sows not just around parturition but also towards weaning, facilitating activity in the nest-leaving phase. The insights gained from this study are further reasons to foster the transition from crated to less restricted farrowing environments. We would like to emphasize that the free-farrowing system used in this study is not ready for practical application without significant improvements in pen design, provision of adequate nesting material, use of sows with maternal traits, and training of animal caretakers. The relatively small sample size used in this study is not suitable for drawing general conclusions about piglet loss rates in open farrowing systems.

## Supplementary Material

skae101_suppl_Supplementary_Materials

## Data Availability

The study’s generated datasets and corresponding analysis code can be obtained from https://doi.org/10.17605/OSF.IO/8SK9V

## Abbreviations

BLMER: Bayesian Linear Mixed-Effects Models
CI: confidence interval
g: g-force (gravitational force equivalent)
ICC: intraclass correlation coefficient
