# Supplementary material for: Sow serenity: automatic long-term measurement of lying behavior in crates and free-farrowing pens using 3D accelerometers
Source: J Anim Sci. 2024 Apr 6;102:skae101. doi: 10.1093/jas/skae101 (PMC11044708; doi:10.1093/jas/skae101)
Supplement: skae101_suppl_Supplementary_Materials [file skae101_suppl_supplementary_materials.pdf]

Sow serenity: automatic long-term measurement of lying behavior in crates and free-farrowing pens using 3D accelerometers

Maximilian Knoll, Lorenz Gygax, Edna Hillmann

Humboldt-Universität zu Berlin, Department of Life Sciences, Albrecht Daniel Thaer Institute of Agricultural and Horticultural Sciences, Animal Husbandry and Ethology, Unter den Linden 6, 10099 Berlin, Germany

Journal of Animal Science.

Supplementary Material: Raw accelerometer data (gray lines), *triact* lying classification (light blue lines), and video analysis lying classification (dark blue lines) for two 24-hour periods per sow, one each in May and June 2022. The dotted line represents the critical lying value of 0.75g. Sow ID 8863 only has data for June because the accelerometer did not record on the day chosen for validation in May.

8118

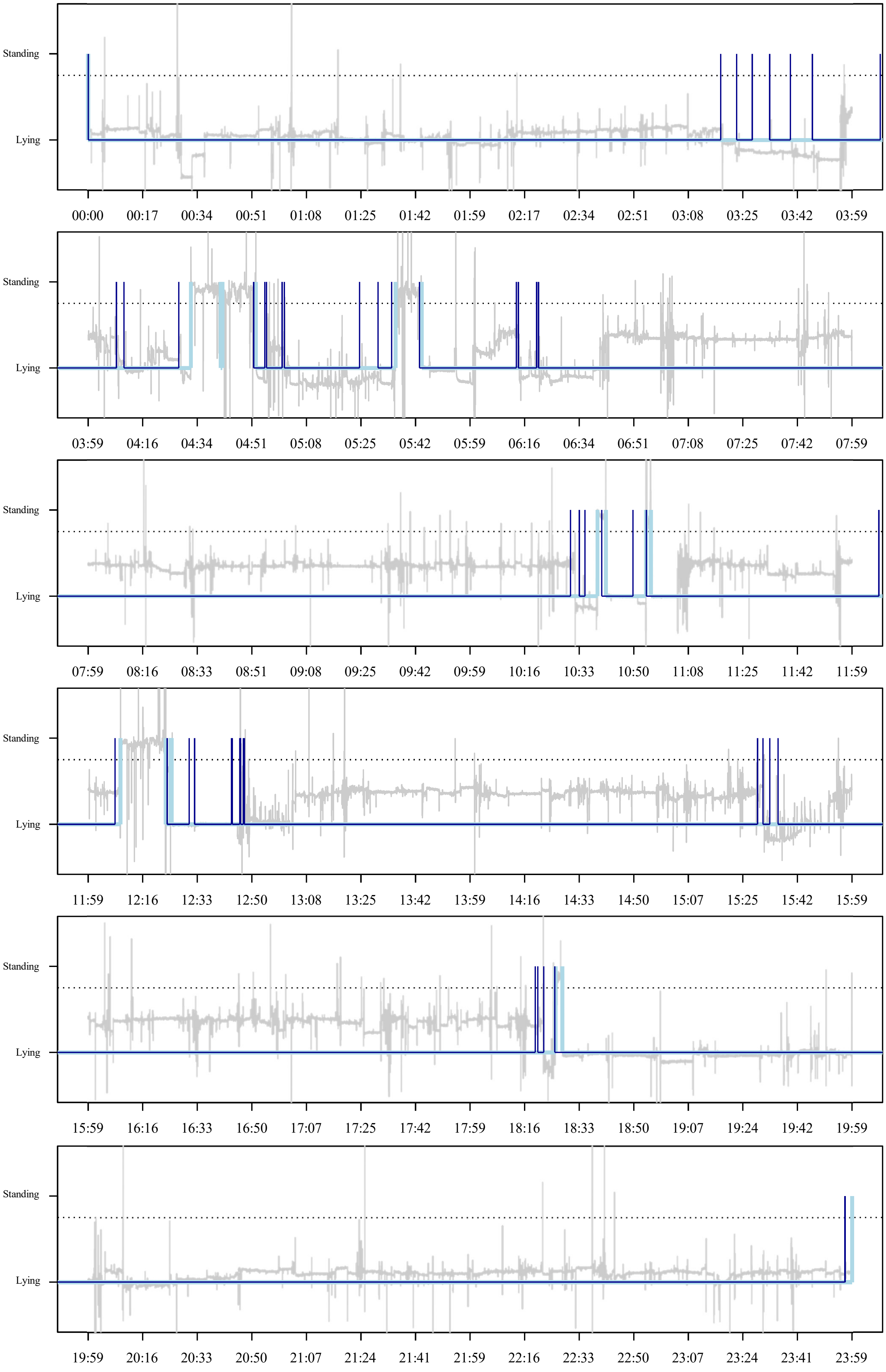

Standing
Lying
00:00 00:17 00:34 00:51 01:08 01:25 01:42 01:59 02:17 02:34 02:51 03:08 03:25 03:42 03:59
03:59 04:16 04:34 04:51 05:08 05:25 05:42 05:59 06:16 06:34 06:51 07:08 07:25 07:42 07:59
07:59 08:16 08:33 08:51 09:08 09:25 09:42 09:59 10:16 10:33 10:50 11:08 11:25 11:42 11:59
11:59 12:16 12:33 12:50 13:08 13:25 13:42 13:59 14:16 14:33 14:50 15:07 15:25 15:42 15:59
15:59 16:16 16:33 16:50 17:07 17:25 17:42 17:59 18:16 18:33 18:50 19:07 19:24 19:42 19:59
19:59 20:16 20:33 20:50 21:07 21:24 21:41 21:59 22:16 22:33 22:50 23:07 23:24 23:41 23:59

8118

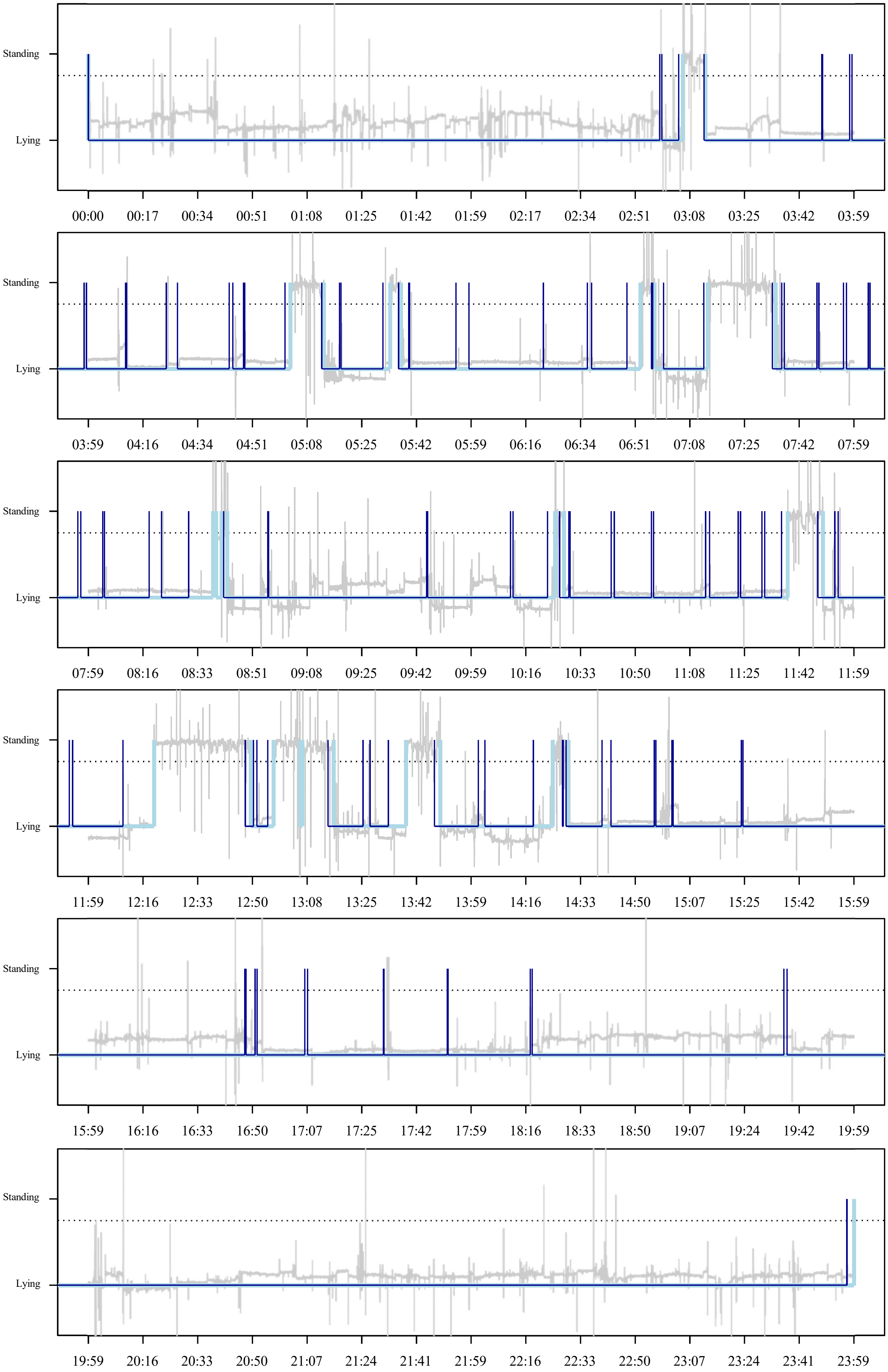

Standing
Lying
00:00 00:17 00:34 00:51 01:08 01:25 01:42 01:59 02:17 02:34 02:51 03:08 03:25 03:42 03:59
03:59 04:16 04:34 04:51 05:08 05:25 05:42 05:59 06:16 06:34 06:51 07:08 07:25 07:42 07:59
07:59 08:16 08:33 08:51 09:08 09:25 09:42 09:59 10:16 10:33 10:50 11:08 11:25 11:42 11:59
11:59 12:16 12:33 12:50 13:08 13:25 13:42 13:59 14:16 14:33 14:50 15:07 15:25 15:42 15:59
15:59 16:16 16:33 16:50 17:07 17:25 17:42 17:59 18:16 18:33 18:50 19:07 19:24 19:42 19:59
19:59 20:16 20:33 20:50 21:07 21:24 21:41 21:59 22:16 22:33 22:50 23:07 23:24 23:41 23:59

9138

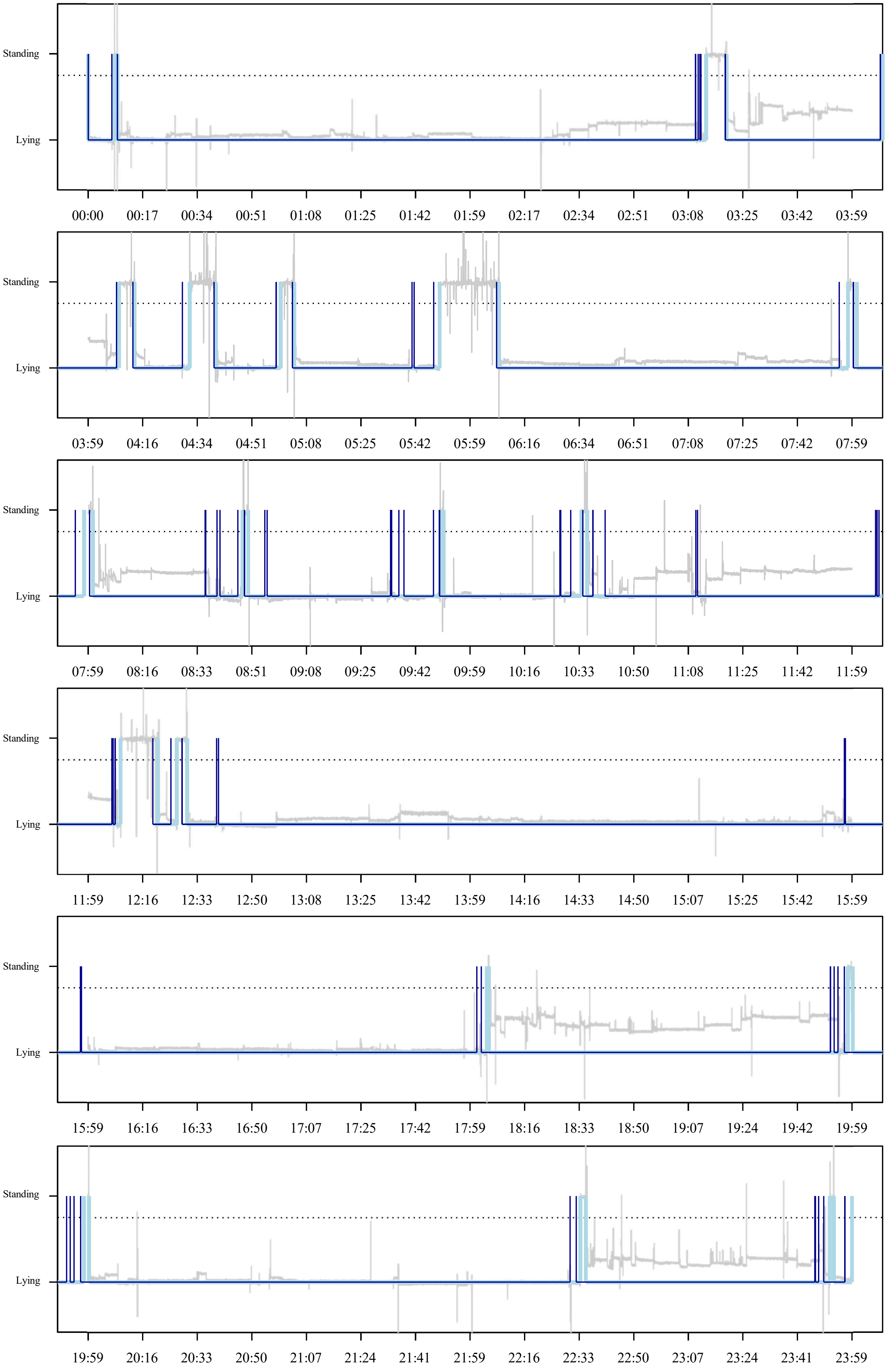
Standing
Lying
00:00 00:17 00:34 00:51 01:08 01:25 01:42 01:59 02:17 02:34 02:51 03:08 03:25 03:42 03:59
03:59 04:16 04:34 04:51 05:08 05:25 05:42 05:59 06:16 06:34 06:51 07:08 07:25 07:42 07:59
07:59 08:16 08:33 08:51 09:08 09:25 09:42 09:59 10:16 10:33 10:50 11:08 11:25 11:42 11:59
11:59 12:16 12:33 12:50 13:08 13:25 13:42 13:59 14:16 14:33 14:50 15:07 15:25 15:42 15:59
15:59 16:16 16:33 16:50 17:07 17:25 17:42 17:59 18:16 18:33 18:50 19:07 19:24 19:42 19:59
19:59 20:16 20:33 20:50 21:07 21:24 21:41 21:59 22:16 22:33 22:50 23:07 23:24 23:41 23:59

9138

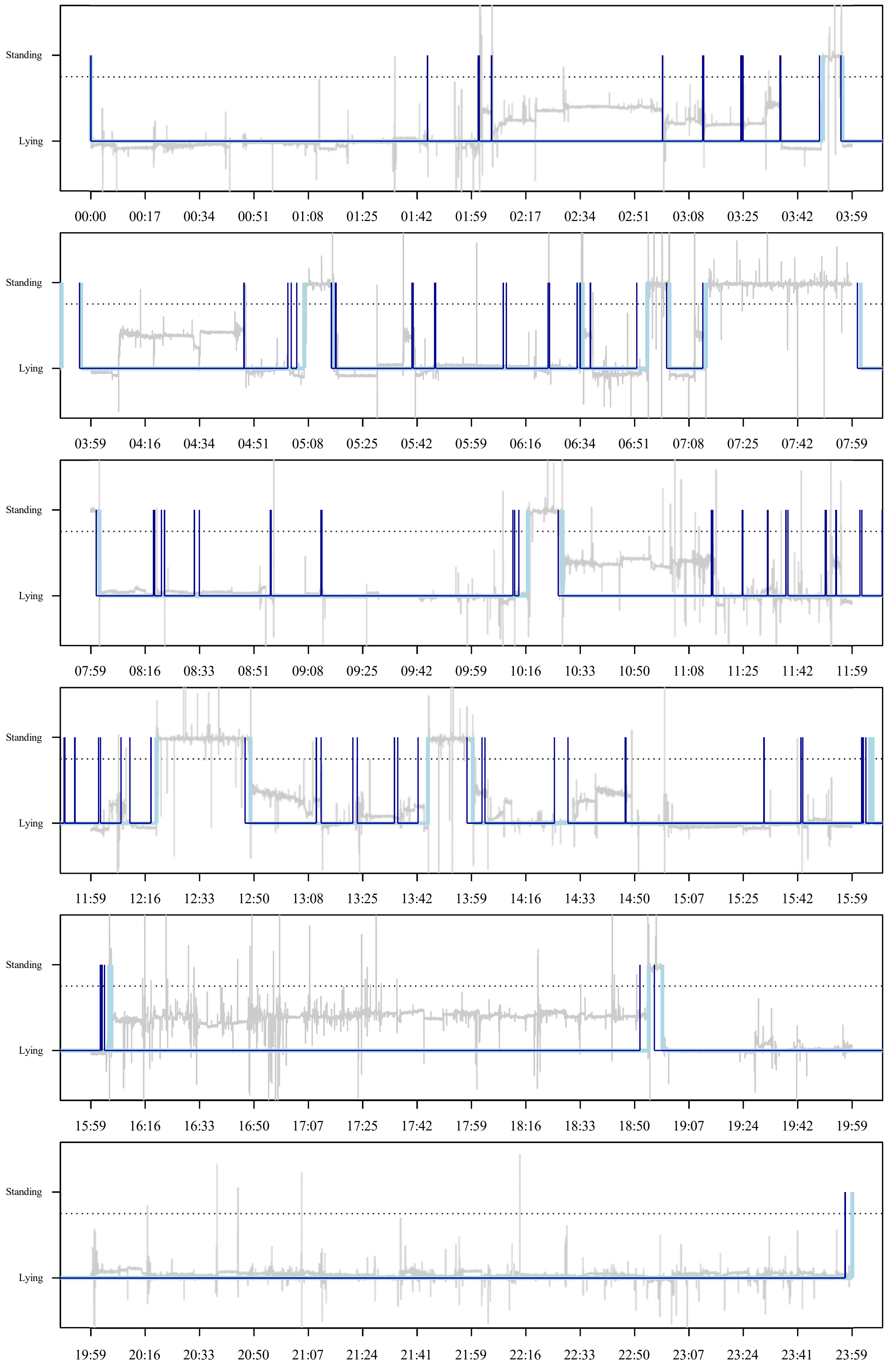
Standing
Lying
00:00
00:17
00:34
00:51
01:08
01:25
01:42
01:59
02:17
02:34
02:51
03:08
03:25
03:42
03:59
04:16
04:34
04:51
05:08
05:25
05:42
05:59
06:16
06:34
06:51
07:08
07:25
07:42
07:59
08:16
08:33
08:51
09:08
09:25
09:42
09:59
10:16
10:33
10:50
11:08
11:25
11:42
11:59
12:16
12:33
12:50
13:08
13:25
13:42
13:59
14:16
14:33
14:50
15:07
15:25
15:42
15:59
16:16
16:33
16:50
17:07
17:25
17:42
17:59
18:16
18:33
18:50
19:07
19:24
19:42
19:59
20:16
20:33
20:50
21:07
21:24
21:41
21:59
22:16
22:33
22:50
23:07
23:24
23:41
23:59

8858

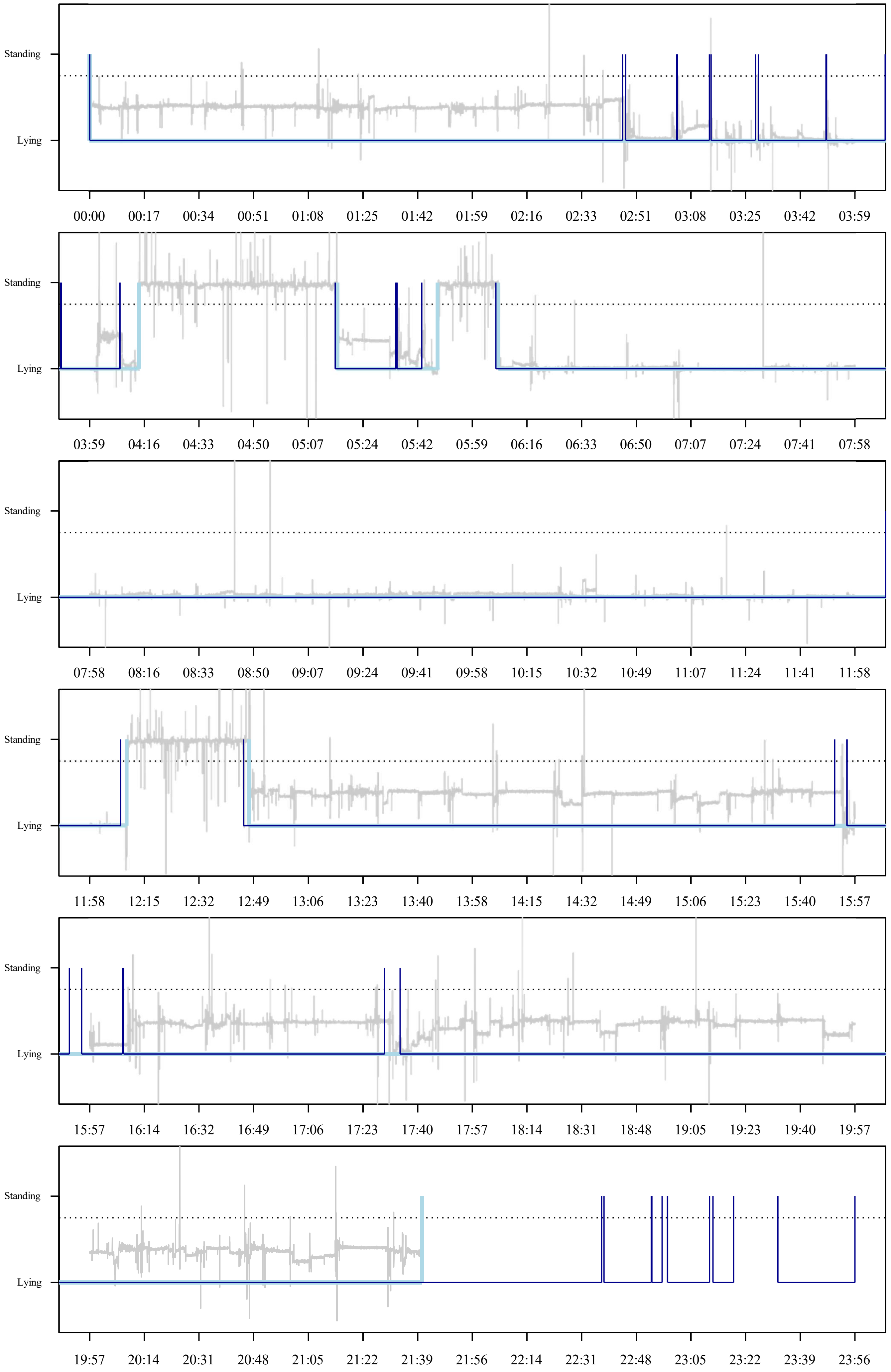
Standing
Lying
00:00 00:17 00:34 00:51 01:08 01:25 01:42 01:59 02:16 02:33 02:51 03:08 03:25 03:42 03:59
03:59 04:16 04:33 04:50 05:07 05:24 05:42 05:59 06:16 06:33 06:50 07:07 07:24 07:41 07:58
07:58 08:16 08:33 08:50 09:07 09:24 09:41 09:58 10:15 10:32 10:49 11:07 11:24 11:41 11:58
11:58 12:15 12:32 12:49 13:06 13:23 13:40 13:58 14:15 14:32 14:49 15:06 15:23 15:40 15:57
15:57 16:14 16:32 16:49 17:06 17:23 17:40 17:57 18:14 18:31 18:48 19:05 19:23 19:40 19:57
19:57 20:14 20:31 20:48 21:05 21:22 21:39 21:56 22:14 22:31 22:48 23:05 23:22 23:39 23:56

8858

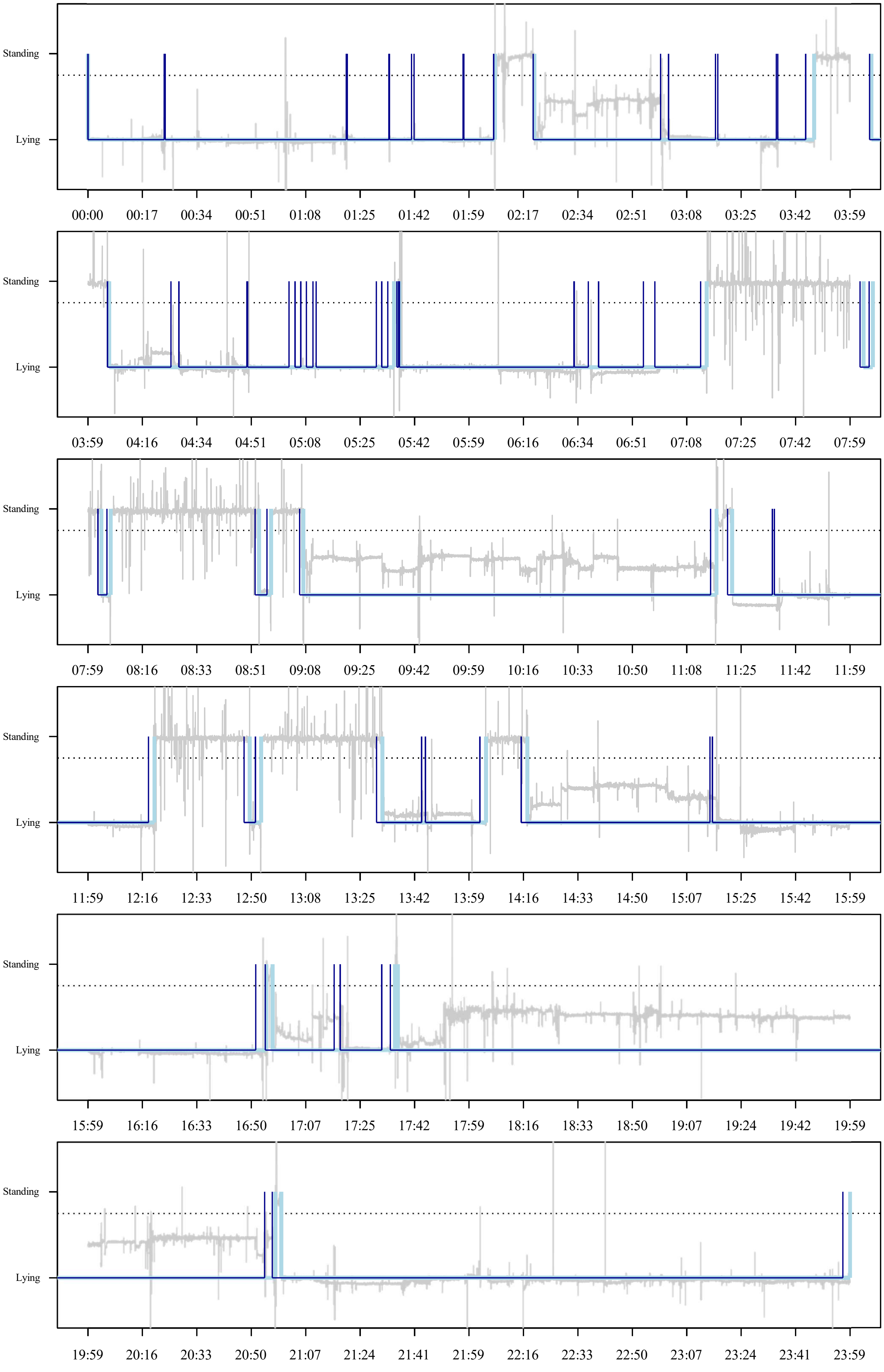
Standing
Lying
00:00 00:17 00:34 00:51 01:08 01:25 01:42 01:59 02:17 02:34 02:51 03:08 03:25 03:42 03:59
03:59 04:16 04:34 04:51 05:08 05:25 05:42 05:59 06:16 06:34 06:51 07:08 07:25 07:42 07:59
07:59 08:16 08:33 08:51 09:08 09:25 09:42 09:59 10:16 10:33 10:50 11:08 11:25 11:42 11:59
11:59 12:16 12:33 12:50 13:08 13:25 13:42 13:59 14:16 14:33 14:50 15:07 15:25 15:42 15:59
15:59 16:16 16:33 16:50 17:07 17:25 17:42 17:59 18:16 18:33 18:50 19:07 19:24 19:42 19:59
19:59 20:16 20:33 20:50 21:07 21:24 21:41 21:59 22:16 22:33 22:50 23:07 23:24 23:41 23:59

8851

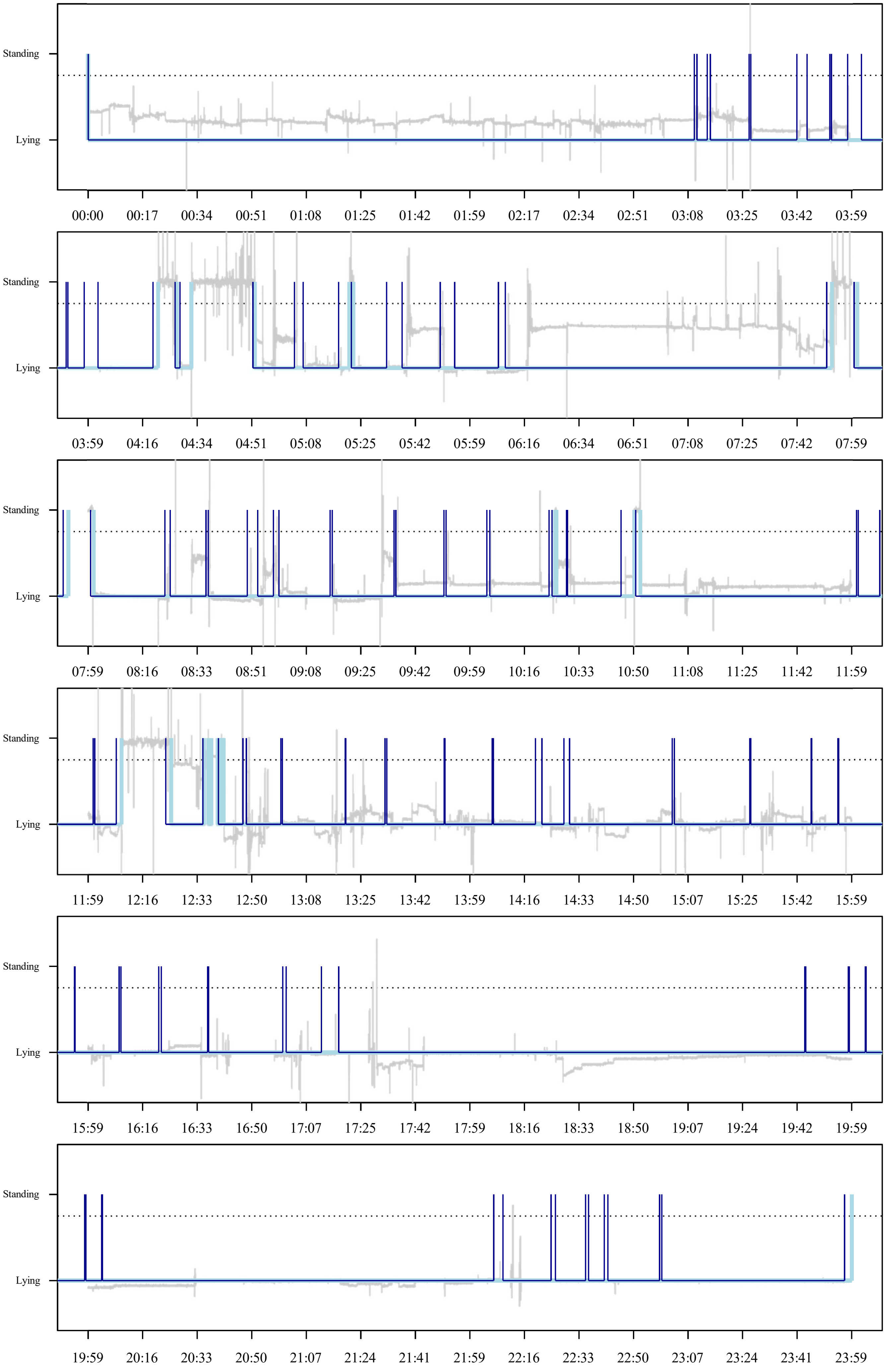
Standing
Lying
00:00 00:17 00:34 00:51 01:08 01:25 01:42 01:59 02:17 02:34 02:51 03:08 03:25 03:42 03:59
03:59 04:16 04:34 04:51 05:08 05:25 05:42 05:59 06:16 06:34 06:51 07:08 07:25 07:42 07:59
07:59 08:16 08:33 08:51 09:08 09:25 09:42 09:59 10:16 10:33 10:50 11:08 11:25 11:42 11:59
11:59 12:16 12:33 12:50 13:08 13:25 13:42 13:59 14:16 14:33 14:50 15:07 15:25 15:42 15:59
15:59 16:16 16:33 16:50 17:07 17:25 17:42 17:59 18:16 18:33 18:50 19:07 19:24 19:42 19:59
19:59 20:16 20:33 20:50 21:07 21:24 21:41 21:59 22:16 22:33 22:50 23:07 23:24 23:41 23:59

8851

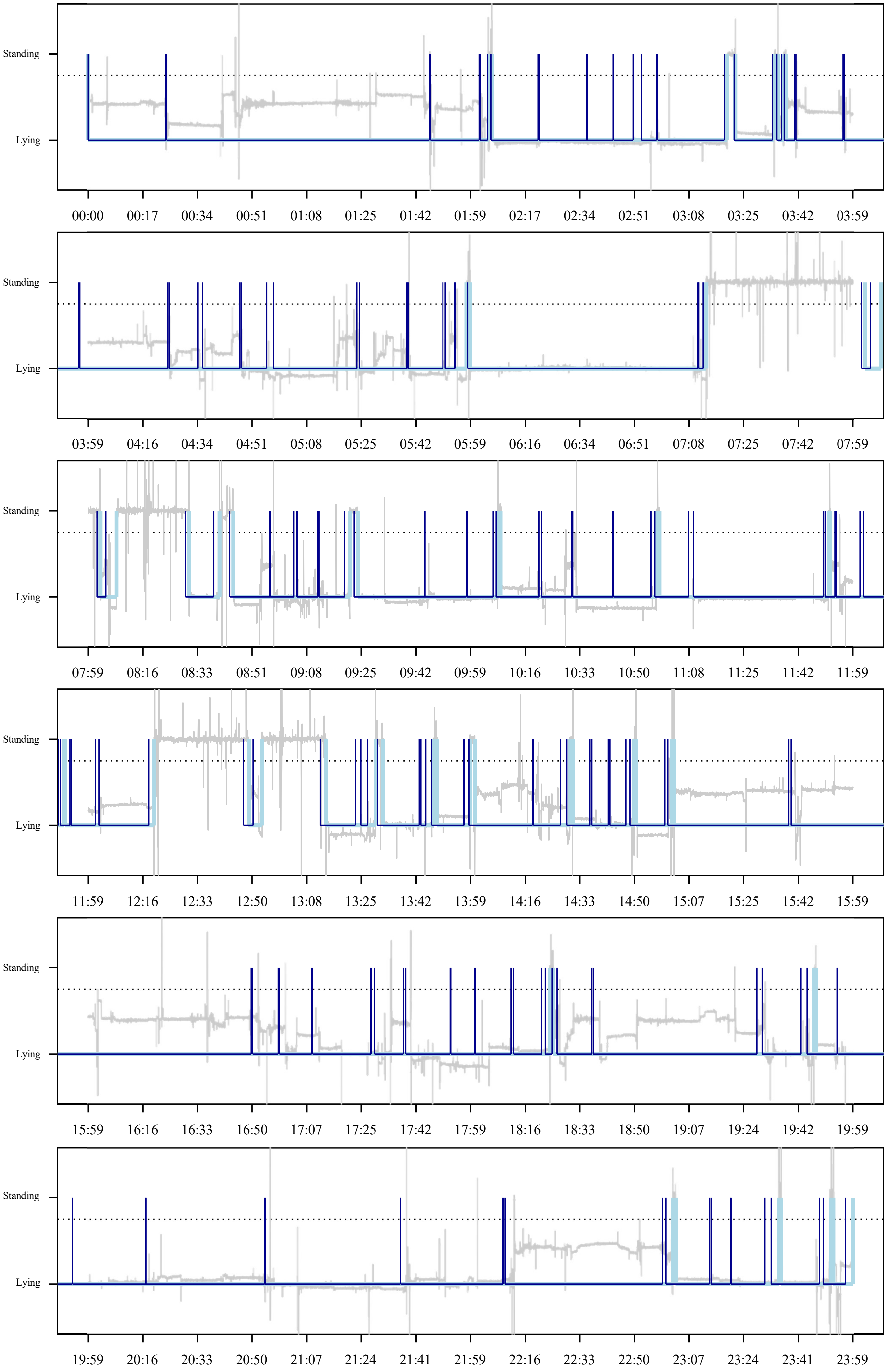
Standing
Lying
00:00 00:17 00:34 00:51 01:08 01:25 01:42 01:59 02:17 02:34 02:51 03:08 03:25 03:42 03:59
Standing
Lying
03:59 04:16 04:34 04:51 05:08 05:25 05:42 05:59 06:16 06:34 06:51 07:08 07:25 07:42 07:59
Standing
Lying
07:59 08:16 08:33 08:51 09:08 09:25 09:42 09:59 10:16 10:33 10:50 11:08 11:25 11:42 11:59
Standing
Lying
11:59 12:16 12:33 12:50 13:08 13:25 13:42 13:59 14:16 14:33 14:50 15:07 15:25 15:42 15:59
Standing
Lying
15:59 16:16 16:33 16:50 17:07 17:25 17:42 17:59 18:16 18:33 18:50 19:07 19:24 19:42 19:59
Standing
Lying
19:59 20:16 20:33 20:50 21:07 21:24 21:41 21:59 22:16 22:33 22:50 23:07 23:24 23:41 23:59

8860

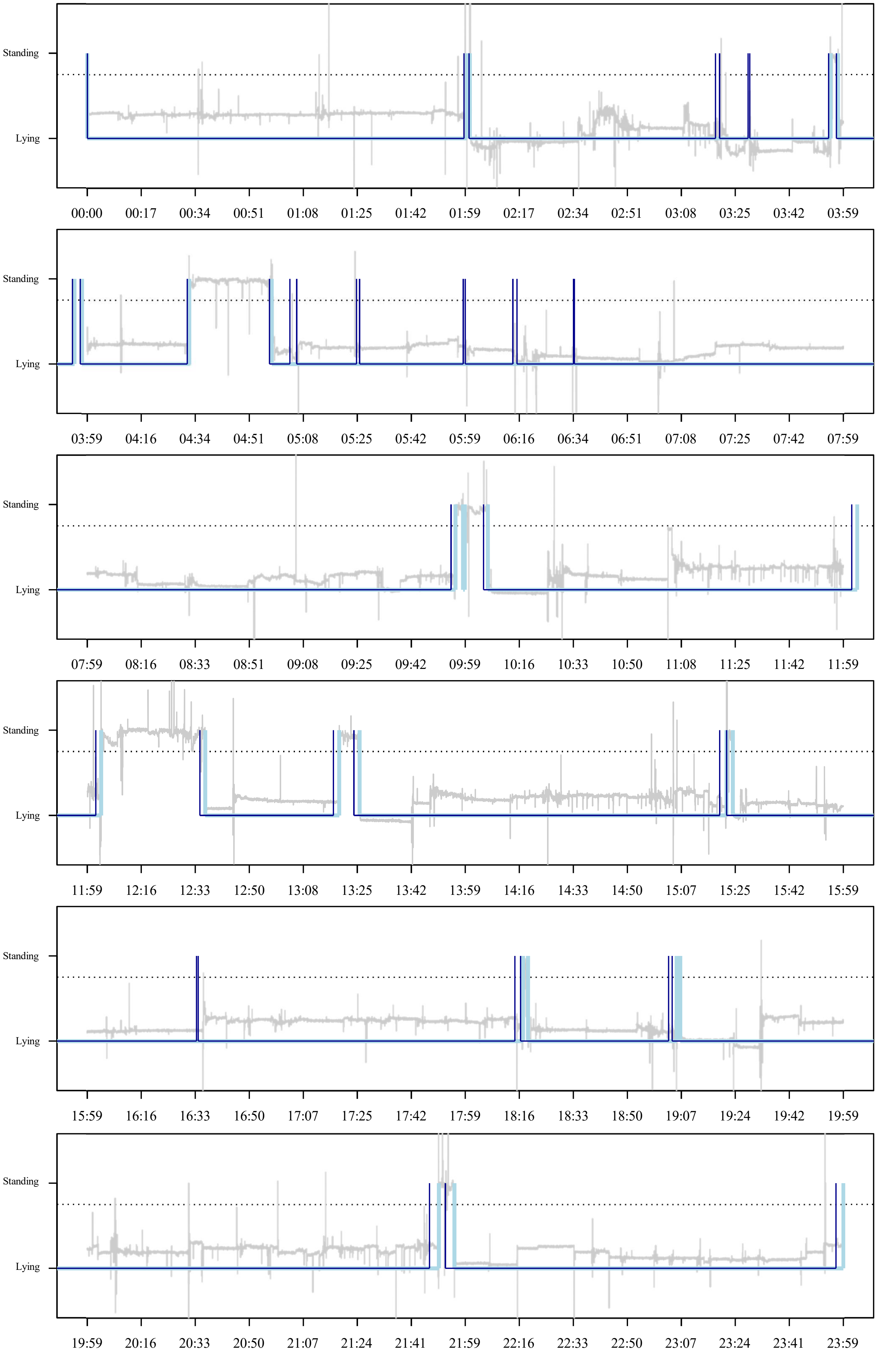
Standing
Lying
00:00 00:17 00:34 00:51 01:08 01:25 01:42 01:59 02:17 02:34 02:51 03:08 03:25 03:42 03:59
Standing
Lying
03:59 04:16 04:34 04:51 05:08 05:25 05:42 05:59 06:16 06:34 06:51 07:08 07:25 07:42 07:59
Standing
Lying
07:59 08:16 08:33 08:51 09:08 09:25 09:42 09:59 10:16 10:33 10:50 11:08 11:25 11:42 11:59
Standing
Lying
11:59 12:16 12:33 12:50 13:08 13:25 13:42 13:59 14:16 14:33 14:50 15:07 15:25 15:42 15:59
Standing
Lying
15:59 16:16 16:33 16:50 17:07 17:25 17:42 17:59 18:16 18:33 18:50 19:07 19:24 19:42 19:59
Standing
Lying
19:59 20:16 20:33 20:50 21:07 21:24 21:41 21:59 22:16 22:33 22:50 23:07 23:24 23:41 23:59

8860

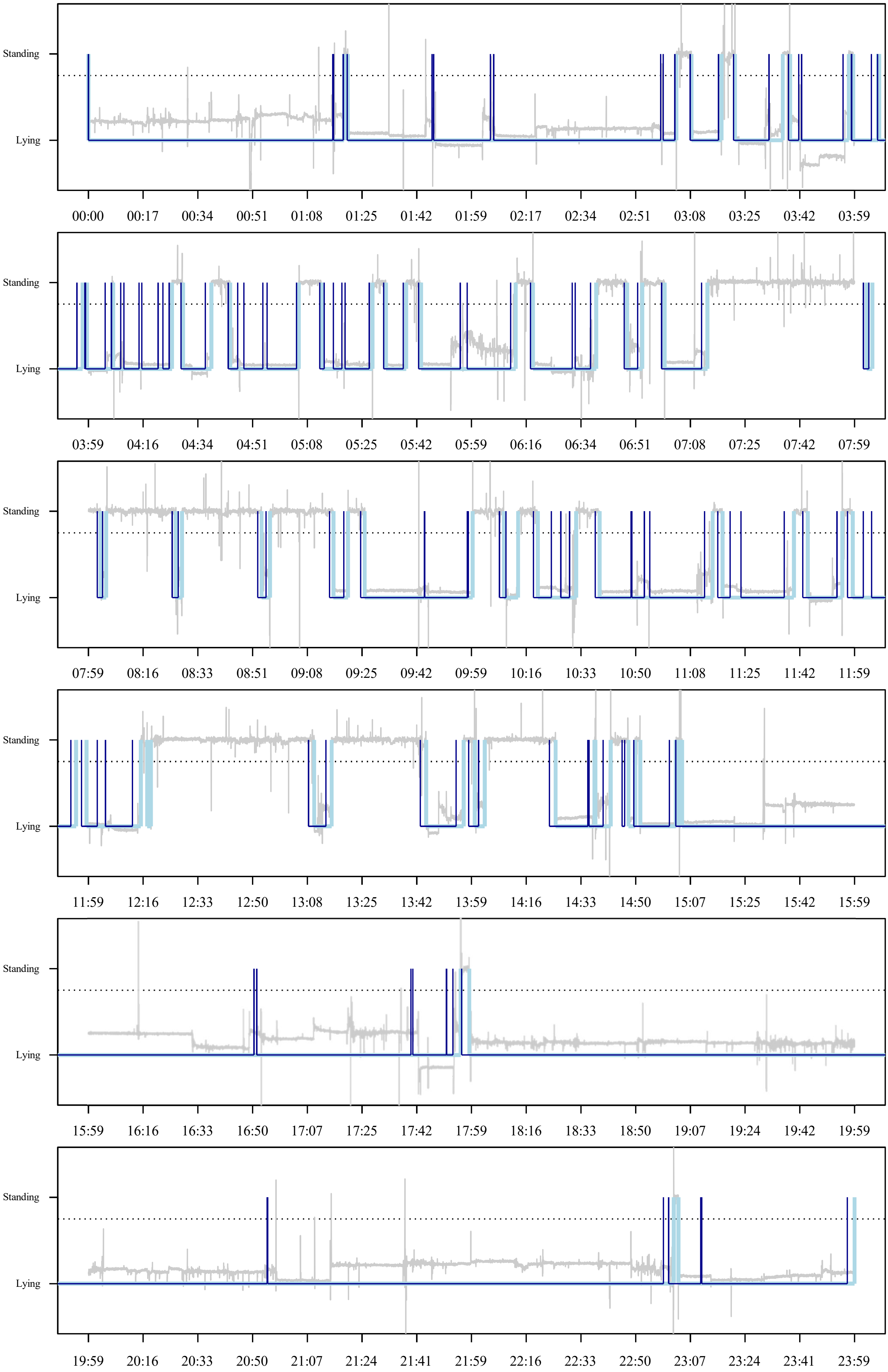
Standing
Lying
00:00 00:17 00:34 00:51 01:08 01:25 01:42 01:59 02:17 02:34 02:51 03:08 03:25 03:42 03:59
Standing
Lying
03:59 04:16 04:34 04:51 05:08 05:25 05:42 05:59 06:16 06:34 06:51 07:08 07:25 07:42 07:59
Standing
Lying
07:59 08:16 08:33 08:51 09:08 09:25 09:42 09:59 10:16 10:33 10:50 11:08 11:25 11:42 11:59
Standing
Lying
11:59 12:16 12:33 12:50 13:08 13:25 13:42 13:59 14:16 14:33 14:50 15:07 15:25 15:42 15:59
Standing
Lying
15:59 16:16 16:33 16:50 17:07 17:25 17:42 17:59 18:16 18:33 18:50 19:07 19:24 19:42 19:59
Standing
Lying
19:59 20:16 20:33 20:50 21:07 21:24 21:41 21:59 22:16 22:33 22:50 23:07 23:24 23:41 23:59

8509

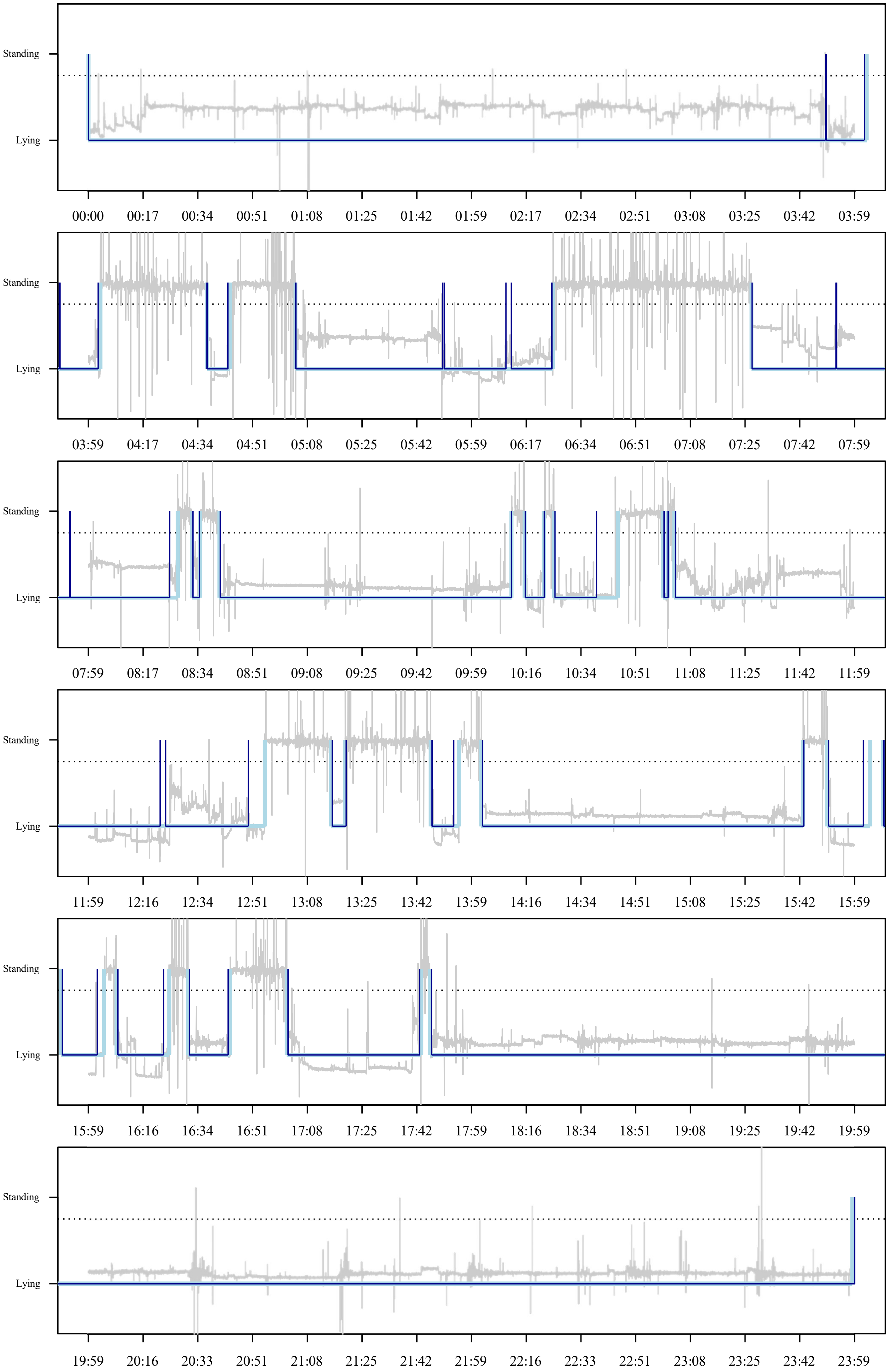

Standing
Lying
00:00 00:17 00:34 00:51 01:08 01:25 01:42 01:59 02:17 02:34 02:51 03:08 03:25 03:42 03:59
03:59 04:17 04:34 04:51 05:08 05:25 05:42 05:59 06:17 06:34 06:51 07:08 07:25 07:42 07:59
07:59 08:17 08:34 08:51 09:08 09:25 09:42 09:59 10:16 10:34 10:51 11:08 11:25 11:42 11:59
11:59 12:16 12:34 12:51 13:08 13:25 13:42 13:59 14:16 14:34 14:51 15:08 15:25 15:42 15:59
15:59 16:16 16:34 16:51 17:08 17:25 17:42 17:59 18:16 18:34 18:51 19:08 19:25 19:42 19:59
19:59 20:16 20:33 20:51 21:08 21:25 21:42 21:59 22:16 22:33 22:51 23:08 23:25 23:42 23:59

8509

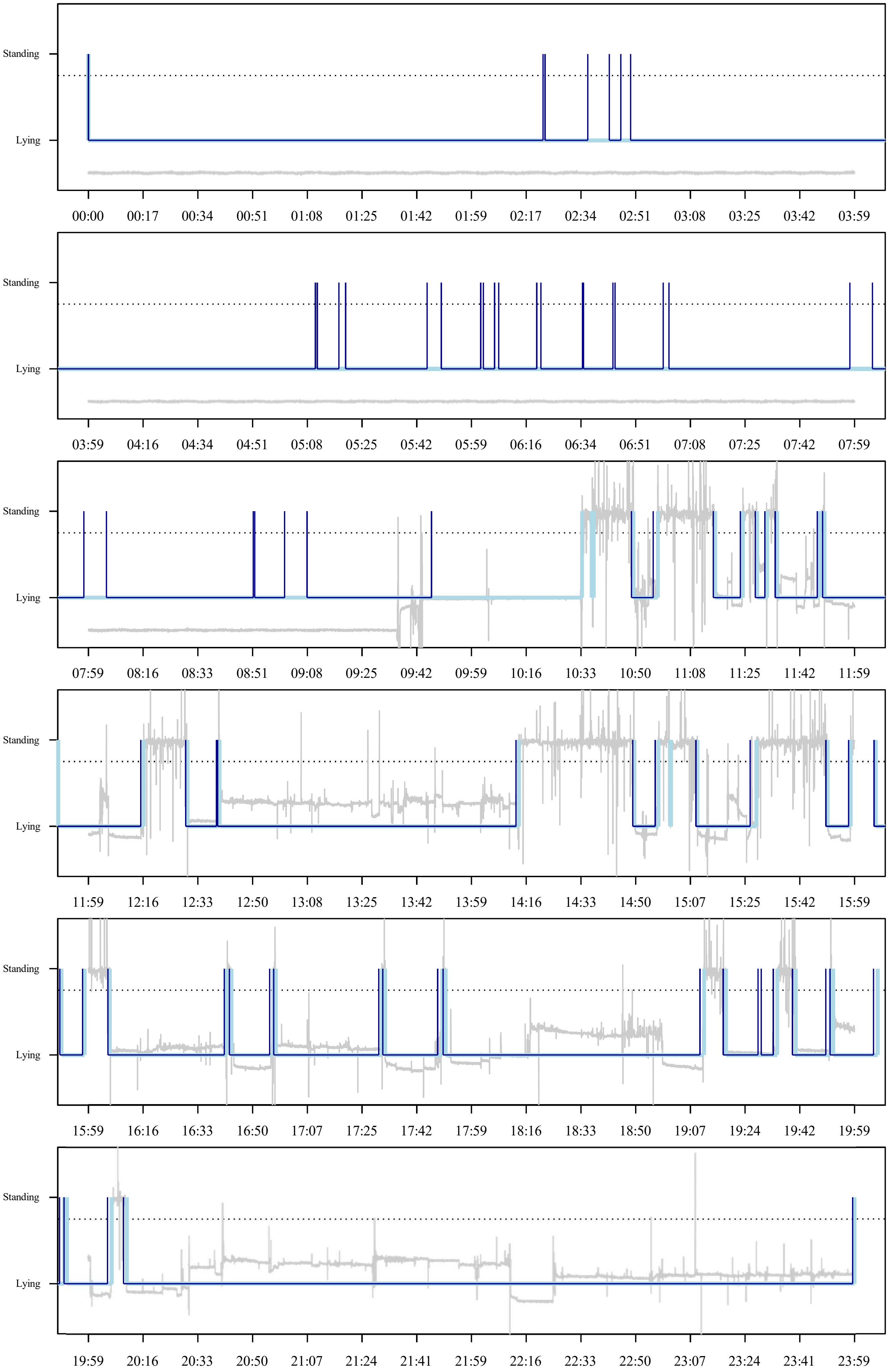

Standing
Lying
00:00 00:17 00:34 00:51 01:08 01:25 01:42 01:59 02:17 02:34 02:51 03:08 03:25 03:42 03:59
03:59 04:16 04:34 04:51 05:08 05:25 05:42 05:59 06:16 06:34 06:51 07:08 07:25 07:42 07:59
07:59 08:16 08:33 08:51 09:08 09:25 09:42 09:59 10:16 10:33 10:50 11:08 11:25 11:42 11:59
11:59 12:16 12:33 12:50 13:08 13:25 13:42 13:59 14:16 14:33 14:50 15:07 15:25 15:42 15:59
15:59 16:16 16:33 16:50 17:07 17:25 17:42 17:59 18:16 18:33 18:50 19:07 19:24 19:42 19:59
19:59 20:16 20:33 20:50 21:07 21:24 21:41 21:59 22:16 22:33 22:50 23:07 23:24 23:41 23:59

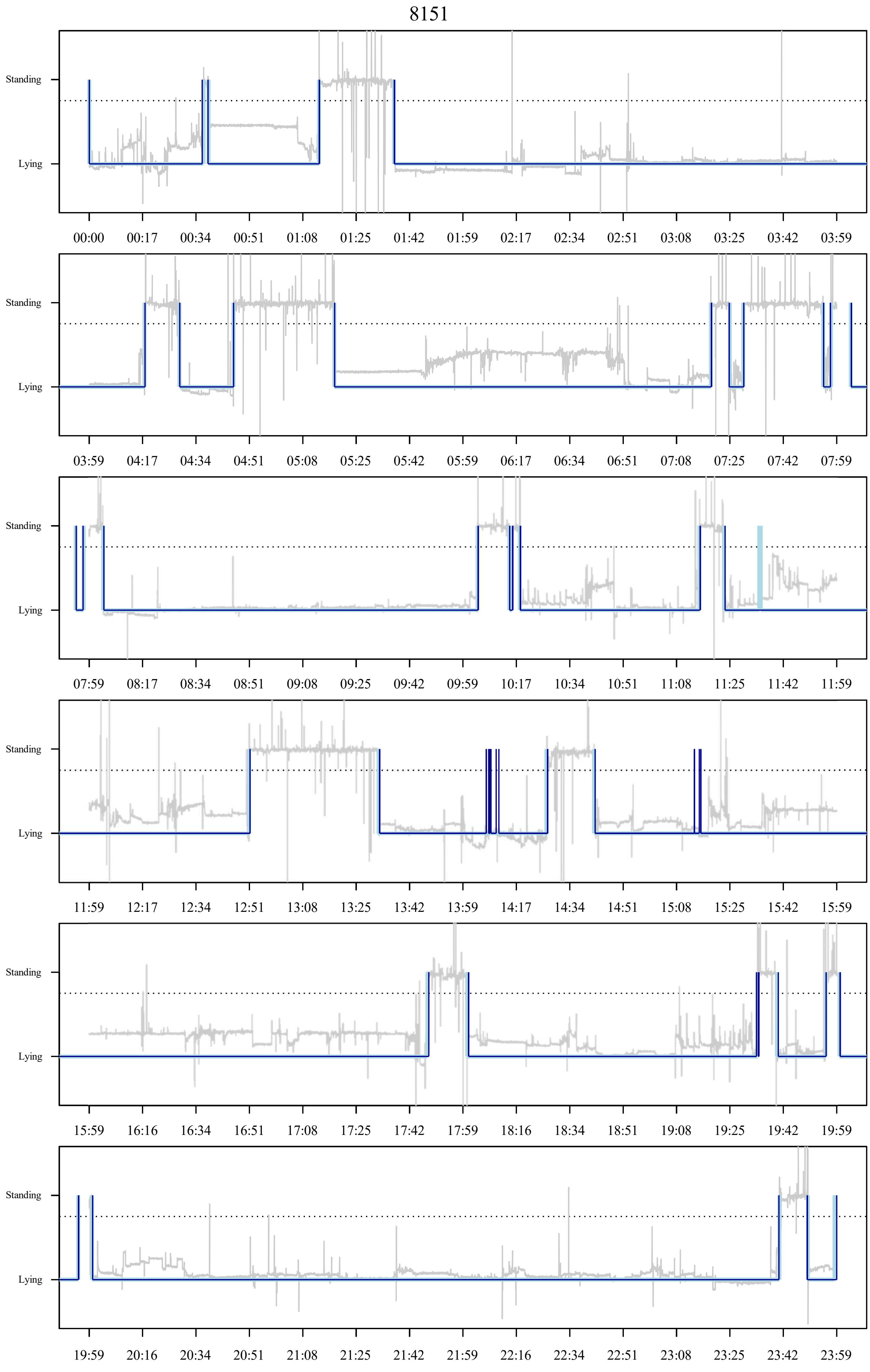

8151
Standing
Lying
00:00 00:17 00:34 00:51 01:08 01:25 01:42 01:59 02:17 02:34 02:51 03:08 03:25 03:42 03:59
03:59 04:17 04:34 04:51 05:08 05:25 05:42 05:59 06:17 06:34 06:51 07:08 07:25 07:42 07:59
07:59 08:17 08:34 08:51 09:08 09:25 09:42 09:59 10:17 10:34 10:51 11:08 11:25 11:42 11:59
11:59 12:17 12:34 12:51 13:08 13:25 13:42 13:59 14:17 14:34 14:51 15:08 15:25 15:42 15:59
15:59 16:16 16:34 16:51 17:08 17:25 17:42 17:59 18:16 18:34 18:51 19:08 19:25 19:42 19:59
19:59 20:16 20:34 20:51 21:08 21:25 21:42 21:59 22:16 22:34 22:51 23:08 23:25 23:42 23:59

8151

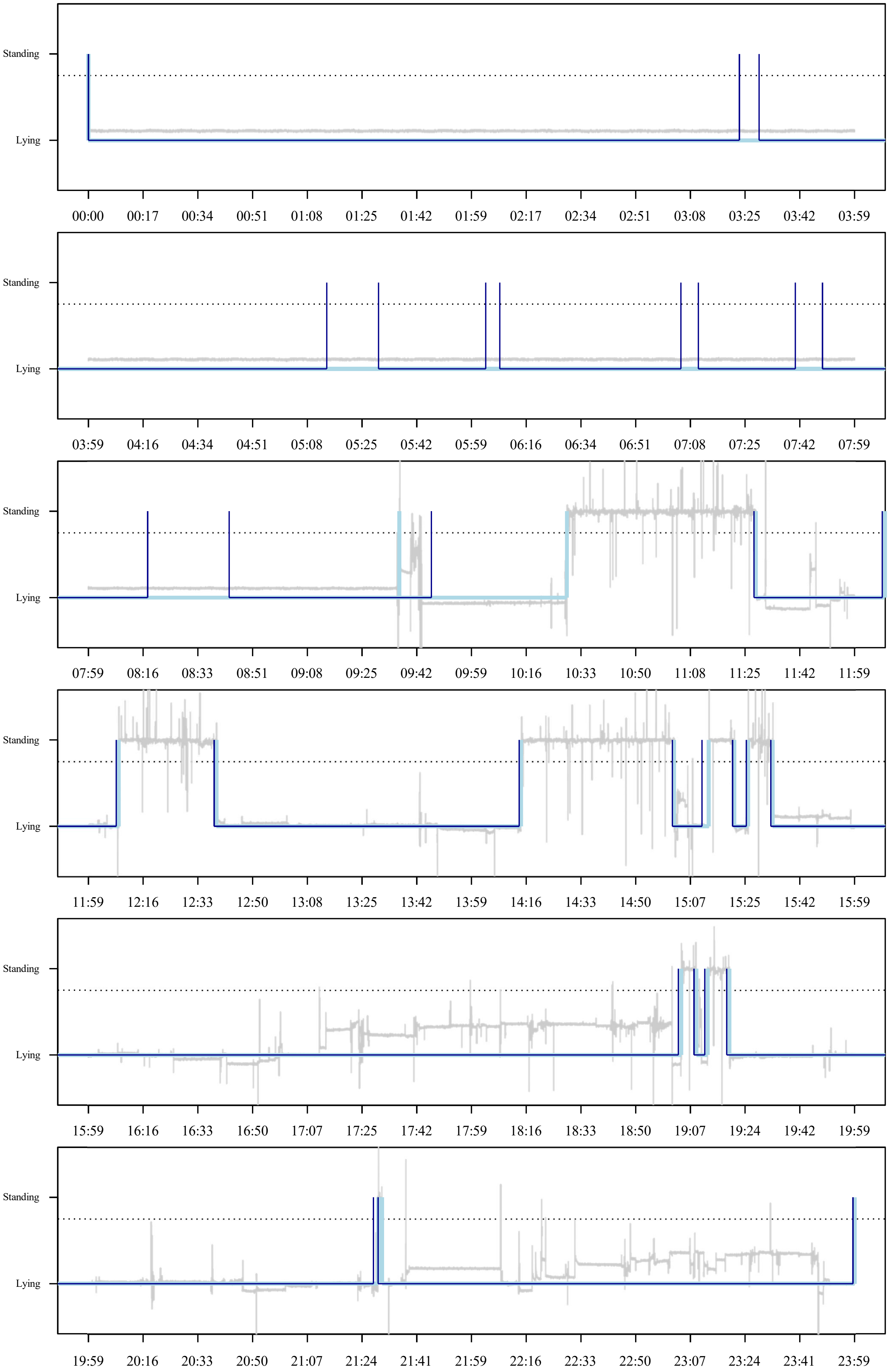

Standing
Lying
00:00 00:17 00:34 00:51 01:08 01:25 01:42 01:59 02:17 02:34 02:51 03:08 03:25 03:42 03:59
03:59 04:16 04:34 04:51 05:08 05:25 05:42 05:59 06:16 06:34 06:51 07:08 07:25 07:42 07:59
07:59 08:16 08:33 08:51 09:08 09:25 09:42 09:59 10:16 10:33 10:50 11:08 11:25 11:42 11:59
11:59 12:16 12:33 12:50 13:08 13:25 13:42 13:59 14:16 14:33 14:50 15:07 15:25 15:42 15:59
15:59 16:16 16:33 16:50 17:07 17:25 17:42 17:59 18:16 18:33 18:50 19:07 19:24 19:42 19:59
19:59 20:16 20:33 20:50 21:07 21:24 21:41 21:59 22:16 22:33 22:50 23:07 23:24 23:41 23:59

8481

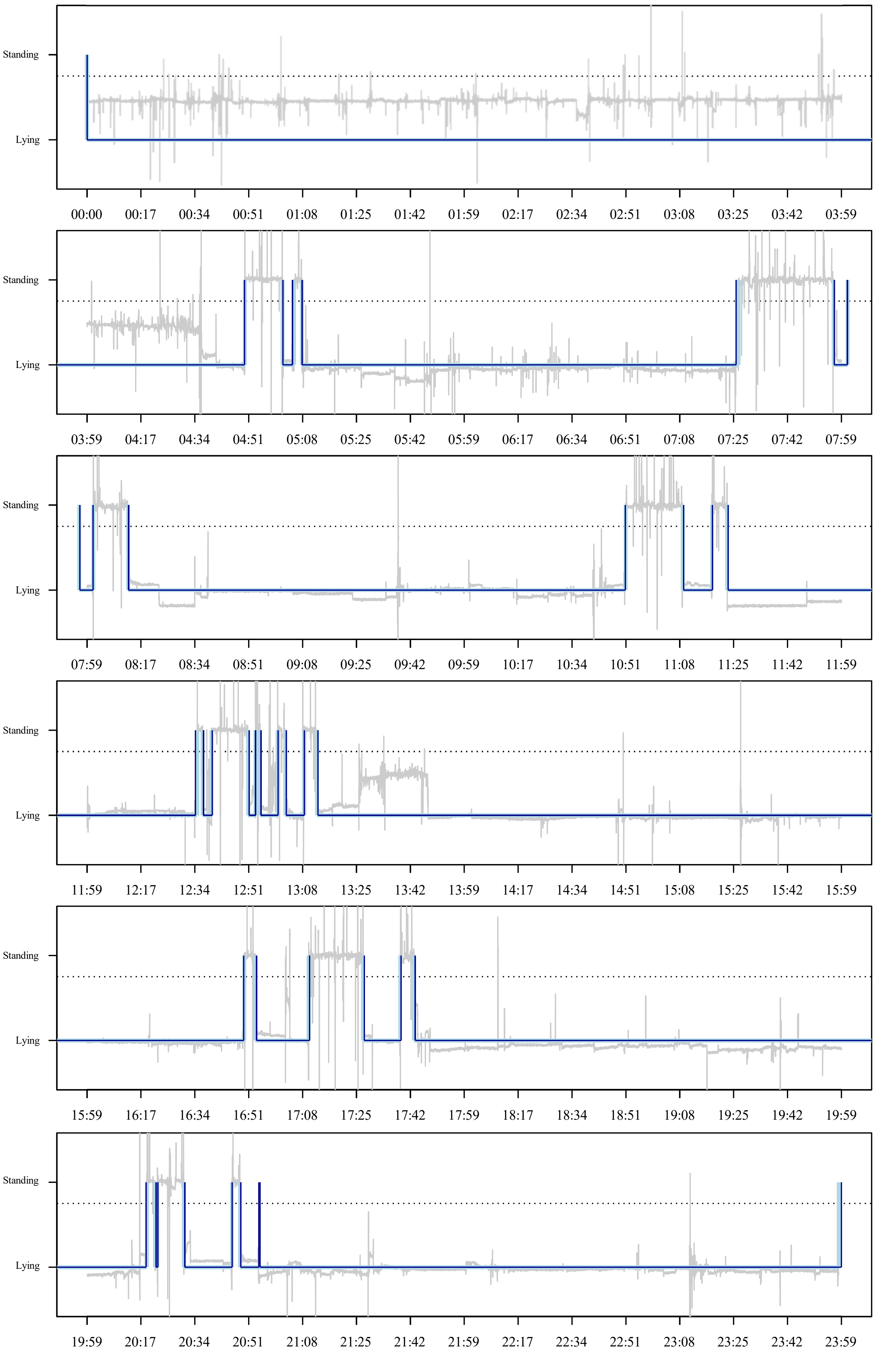
Standing
Lying
00:00
00:17
00:34
00:51
01:08
01:25
01:42
01:59
02:17
02:34
02:51
03:08
03:25
03:42
03:59
04:17
04:34
04:51
05:08
05:25
05:42
05:59
06:17
06:34
06:51
07:08
07:25
07:42
07:59
08:17
08:34
08:51
09:08
09:25
09:42
09:59
10:17
10:34
10:51
11:08
11:25
11:42
11:59
12:17
12:34
12:51
13:08
13:25
13:42
13:59
14:17
14:34
14:51
15:08
15:25
15:42
15:59
16:17
16:34
16:51
17:08
17:25
17:42
17:59
18:17
18:34
18:51
19:08
19:25
19:42
19:59
20:17
20:34
20:51
21:08
21:25
21:42
21:59
22:17
22:34
22:51
23:08
23:25
23:42
23:59

8481

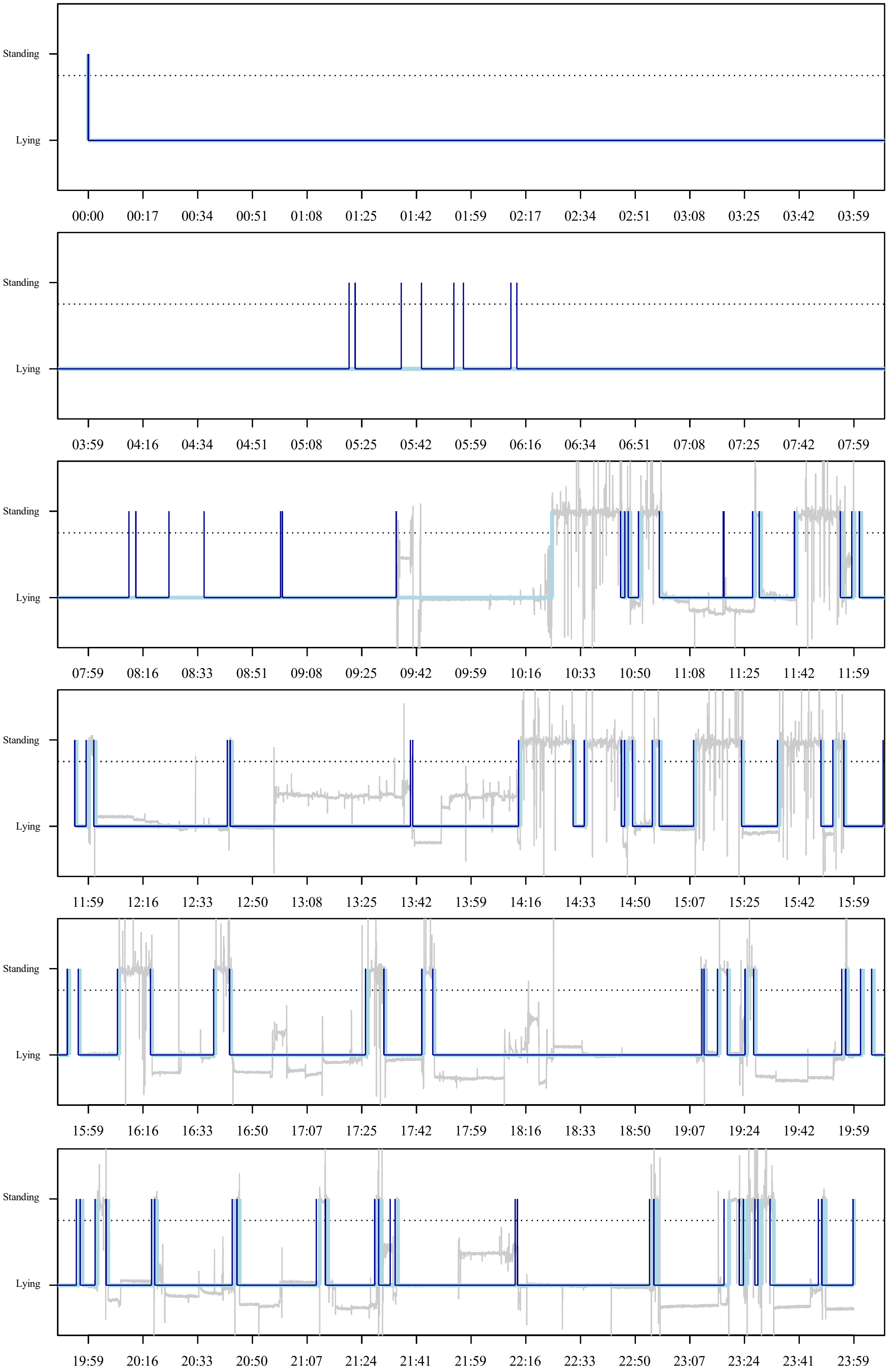
Standing
Lying
00:00 00:17 00:34 00:51 01:08 01:25 01:42 01:59 02:17 02:34 02:51 03:08 03:25 03:42 03:59
03:59 04:16 04:34 04:51 05:08 05:25 05:42 05:59 06:16 06:34 06:51 07:08 07:25 07:42 07:59
07:59 08:16 08:33 08:51 09:08 09:25 09:42 09:59 10:16 10:33 10:50 11:08 11:25 11:42 11:59
11:59 12:16 12:33 12:50 13:08 13:25 13:42 13:59 14:16 14:33 14:50 15:07 15:25 15:42 15:59
15:59 16:16 16:33 16:50 17:07 17:25 17:42 17:59 18:16 18:33 18:50 19:07 19:24 19:42 19:59
19:59 20:16 20:33 20:50 21:07 21:24 21:41 21:59 22:16 22:33 22:50 23:07 23:24 23:41 23:59

0152

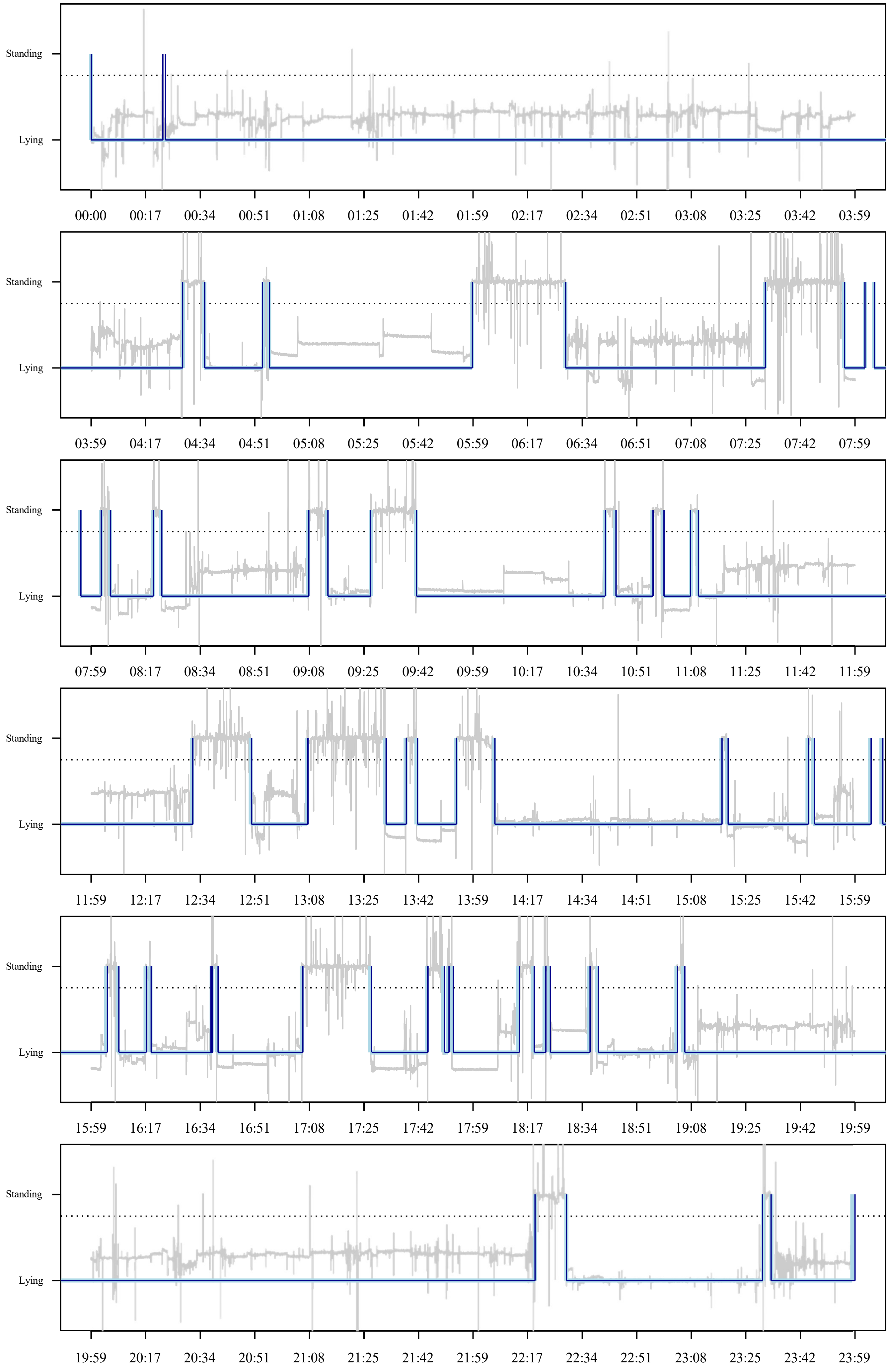
Standing
Lying
00:00 00:17 00:34 00:51 01:08 01:25 01:42 01:59 02:17 02:34 02:51 03:08 03:25 03:42 03:59
Standing
Lying
03:59 04:17 04:34 04:51 05:08 05:25 05:42 05:59 06:17 06:34 06:51 07:08 07:25 07:42 07:59
Standing
Lying
07:59 08:17 08:34 08:51 09:08 09:25 09:42 09:59 10:17 10:34 10:51 11:08 11:25 11:42 11:59
Standing
Lying
11:59 12:17 12:34 12:51 13:08 13:25 13:42 13:59 14:17 14:34 14:51 15:08 15:25 15:42 15:59
Standing
Lying
15:59 16:17 16:34 16:51 17:08 17:25 17:42 17:59 18:17 18:34 18:51 19:08 19:25 19:42 19:59
Standing
Lying
19:59 20:17 20:34 20:51 21:08 21:25 21:42 21:59 22:17 22:34 22:51 23:08 23:25 23:42 23:59

0152

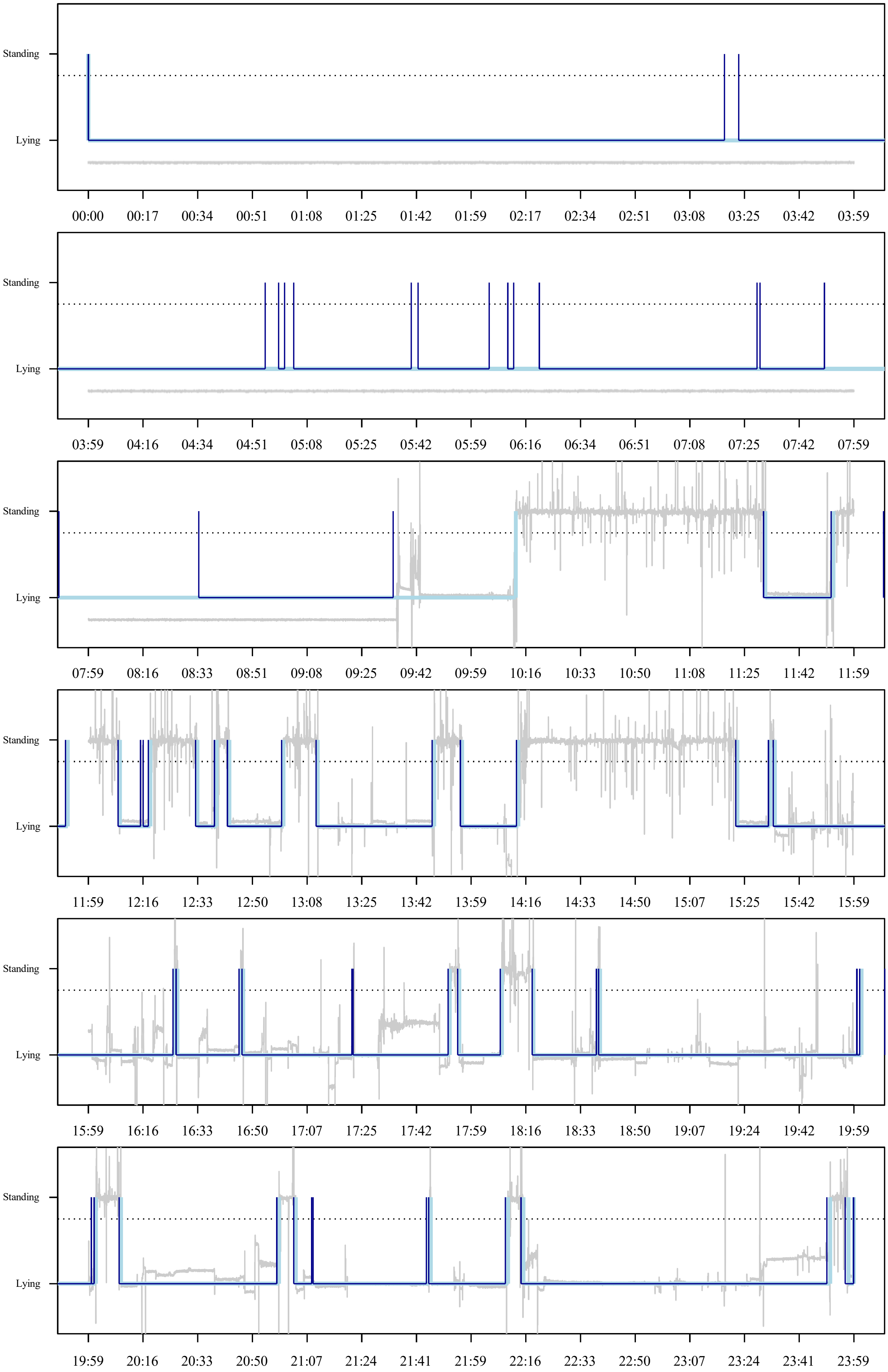
Standing
Lying
00:00 00:17 00:34 00:51 01:08 01:25 01:42 01:59 02:17 02:34 02:51 03:08 03:25 03:42 03:59
03:59 04:16 04:34 04:51 05:08 05:25 05:42 05:59 06:16 06:34 06:51 07:08 07:25 07:42 07:59
07:59 08:16 08:33 08:51 09:08 09:25 09:42 09:59 10:16 10:33 10:50 11:08 11:25 11:42 11:59
11:59 12:16 12:33 12:50 13:08 13:25 13:42 13:59 14:16 14:33 14:50 15:07 15:25 15:42 15:59
15:59 16:16 16:33 16:50 17:07 17:25 17:42 17:59 18:16 18:33 18:50 19:07 19:24 19:42 19:59
19:59 20:16 20:33 20:50 21:07 21:24 21:41 21:59 22:16 22:33 22:50 23:07 23:24 23:41 23:59

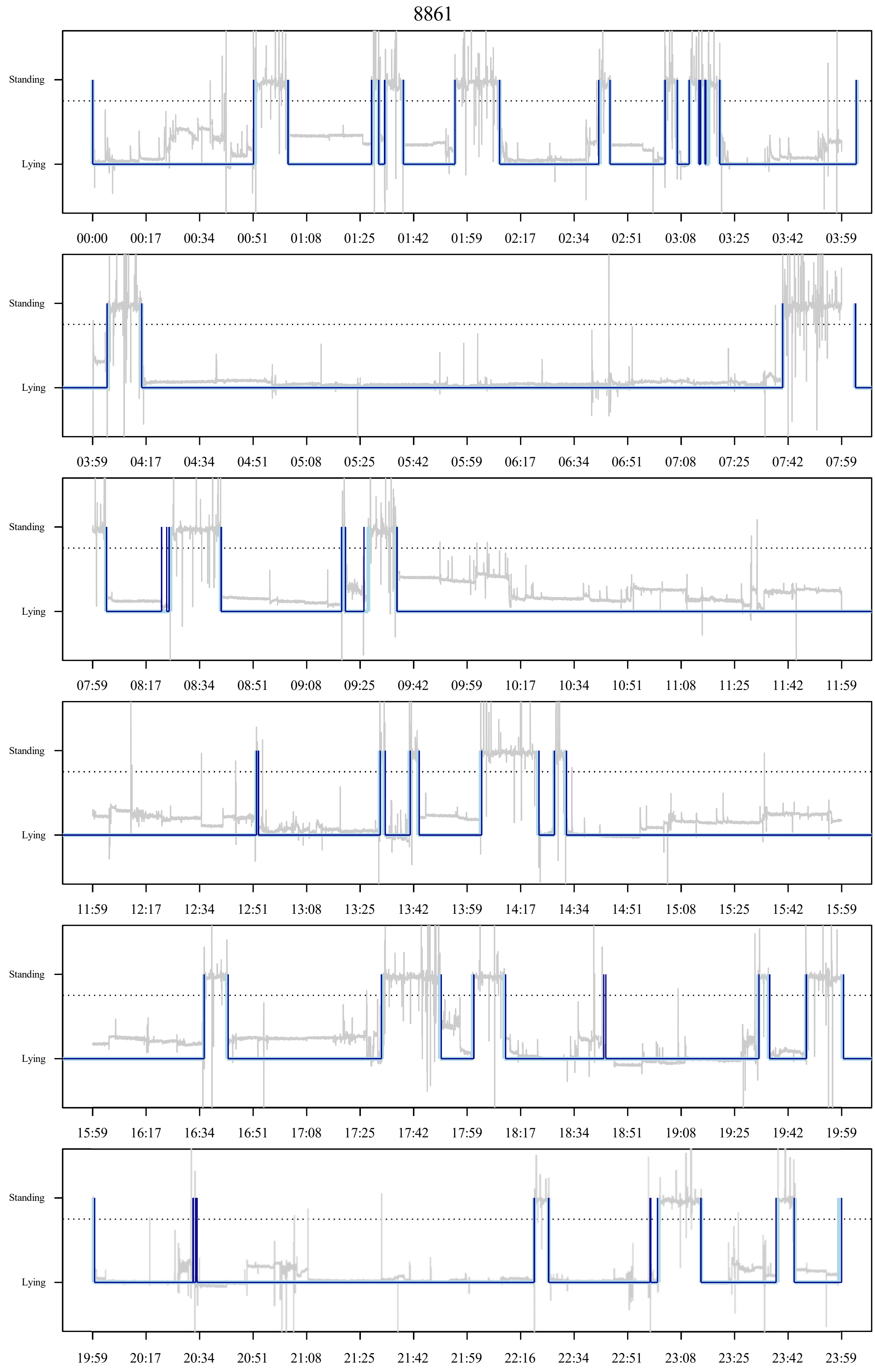
8861
Standing
Lying
00:00
00:17
00:34
00:51
01:08
01:25
01:42
01:59
02:17
02:34
02:51
03:08
03:25
03:42
03:59
04:17
04:34
04:51
05:08
05:25
05:42
05:59
06:17
06:34
06:51
07:08
07:25
07:42
07:59
08:17
08:34
08:51
09:08
09:25
09:42
09:59
10:17
10:34
10:51
11:08
11:25
11:42
11:59
12:17
12:34
12:51
13:08
13:25
13:42
13:59
14:17
14:34
14:51
15:08
15:25
15:42
15:59
16:17
16:34
16:51
17:08
17:25
17:42
17:59
18:17
18:34
18:51
19:08
19:25
19:42
19:59
20:17
20:34
20:51
21:08
21:25
21:42
21:59
22:16
22:34
22:51
23:08
23:25
23:42
23:59

8861

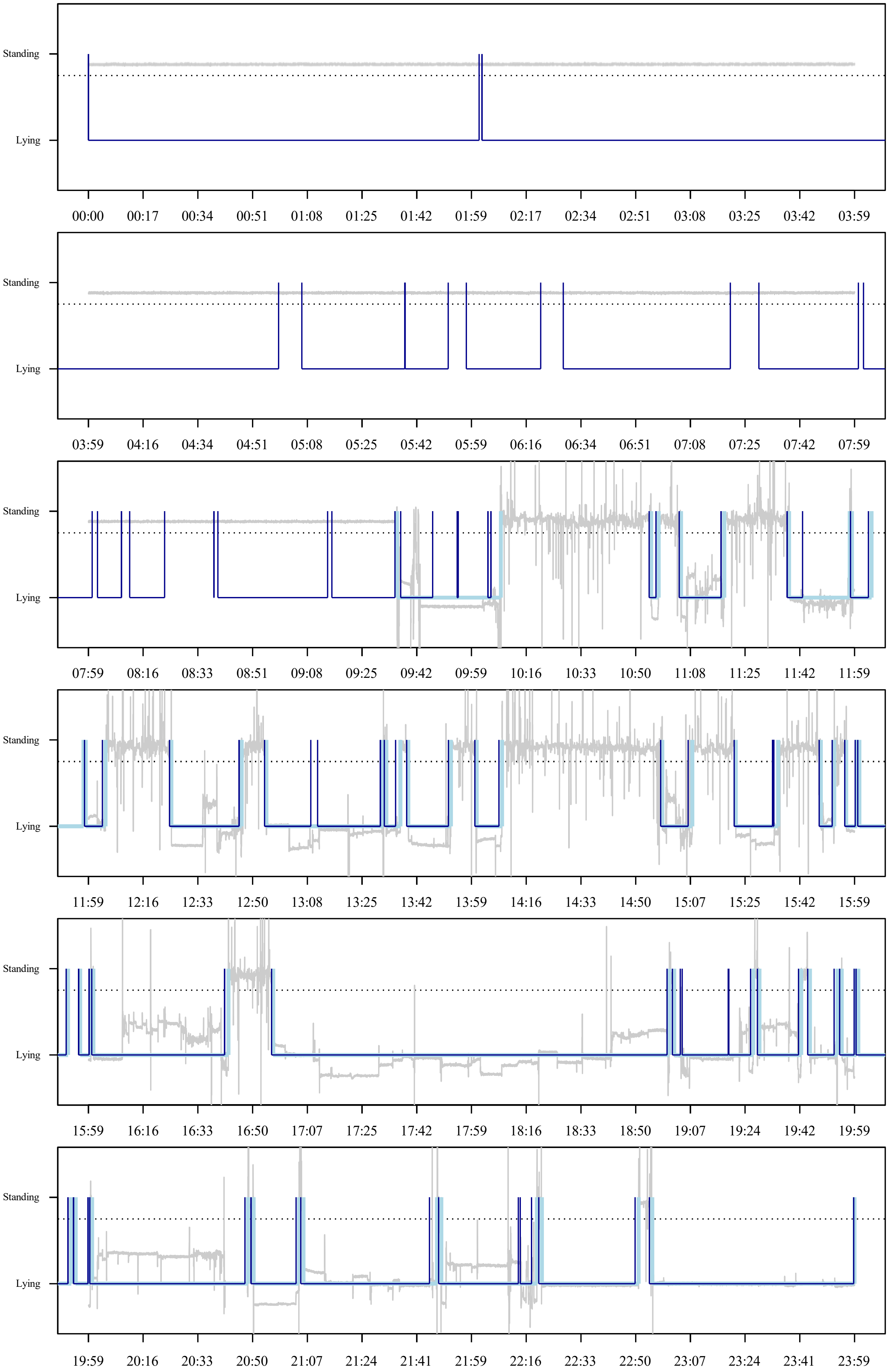
Standing
Lying
00:00
00:17
00:34
00:51
01:08
01:25
01:42
01:59
02:17
02:34
02:51
03:08
03:25
03:42
03:59
Standing
Lying
03:59
04:16
04:34
04:51
05:08
05:25
05:42
05:59
06:16
06:34
06:51
07:08
07:25
07:42
07:59
Standing
Lying
07:59
08:16
08:33
08:51
09:08
09:25
09:42
09:59
10:16
10:33
10:50
11:08
11:25
11:42
11:59
Standing
Lying
11:59
12:16
12:33
12:50
13:08
13:25
13:42
13:59
14:16
14:33
14:50
15:07
15:25
15:42
15:59
Standing
Lying
15:59
16:16
16:33
16:50
17:07
17:25
17:42
17:59
18:16
18:33
18:50
19:07
19:24
19:42
19:59
Standing
Lying
19:59
20:16
20:33
20:50
21:07
21:24
21:41
21:59
22:16
22:33
22:50
23:07
23:24
23:41
23:59

8854

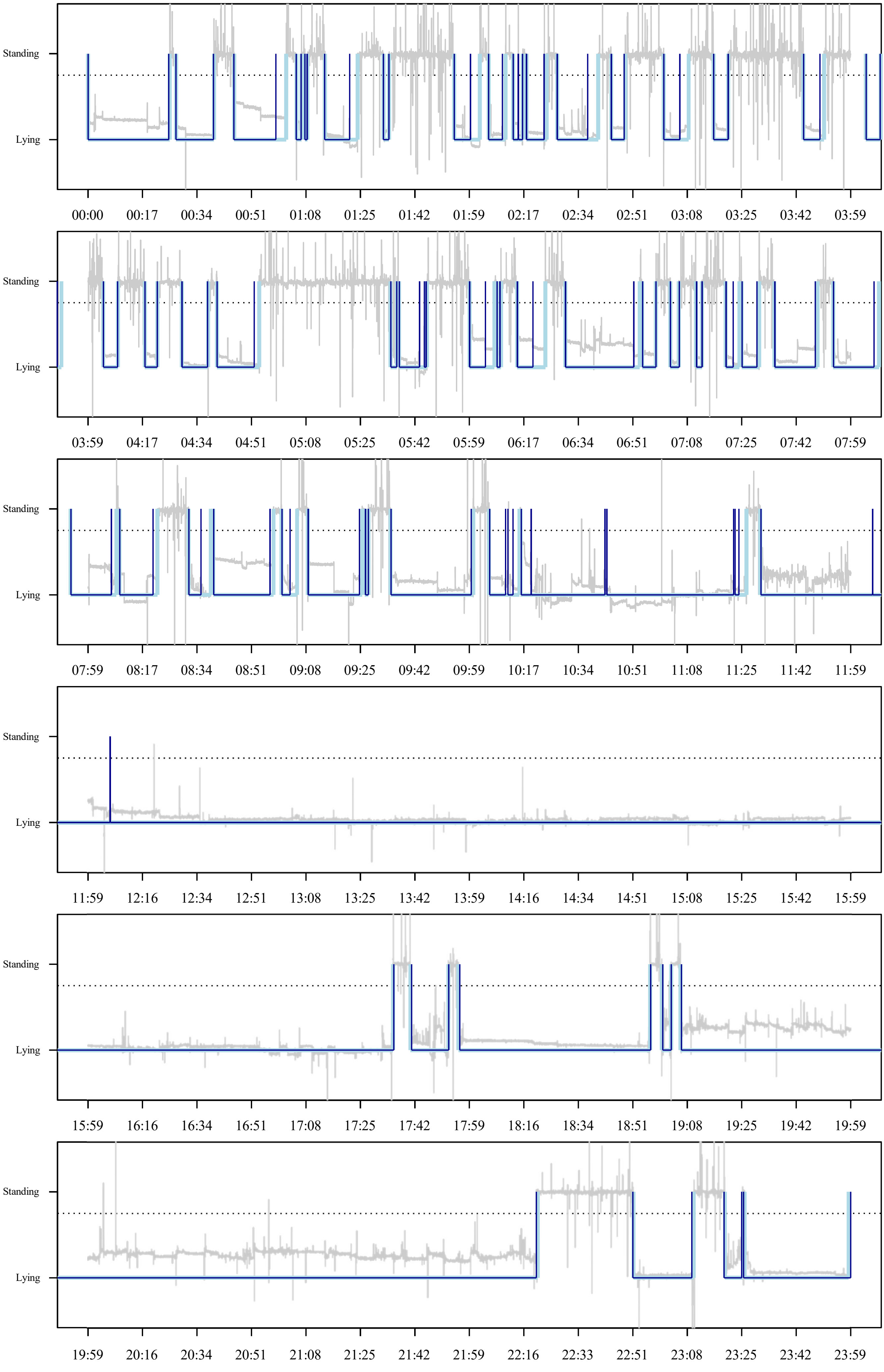
Standing
Lying
00:00 00:17 00:34 00:51 01:08 01:25 01:42 01:59 02:17 02:34 02:51 03:08 03:25 03:42 03:59
03:59 04:17 04:34 04:51 05:08 05:25 05:42 05:59 06:17 06:34 06:51 07:08 07:25 07:42 07:59
07:59 08:17 08:34 08:51 09:08 09:25 09:42 09:59 10:17 10:34 10:51 11:08 11:25 11:42 11:59
11:59 12:16 12:34 12:51 13:08 13:25 13:42 13:59 14:16 14:34 14:51 15:08 15:25 15:42 15:59
15:59 16:16 16:34 16:51 17:08 17:25 17:42 17:59 18:16 18:34 18:51 19:08 19:25 19:42 19:59
19:59 20:16 20:34 20:51 21:08 21:25 21:42 21:59 22:16 22:33 22:51 23:08 23:25 23:42 23:59

8854

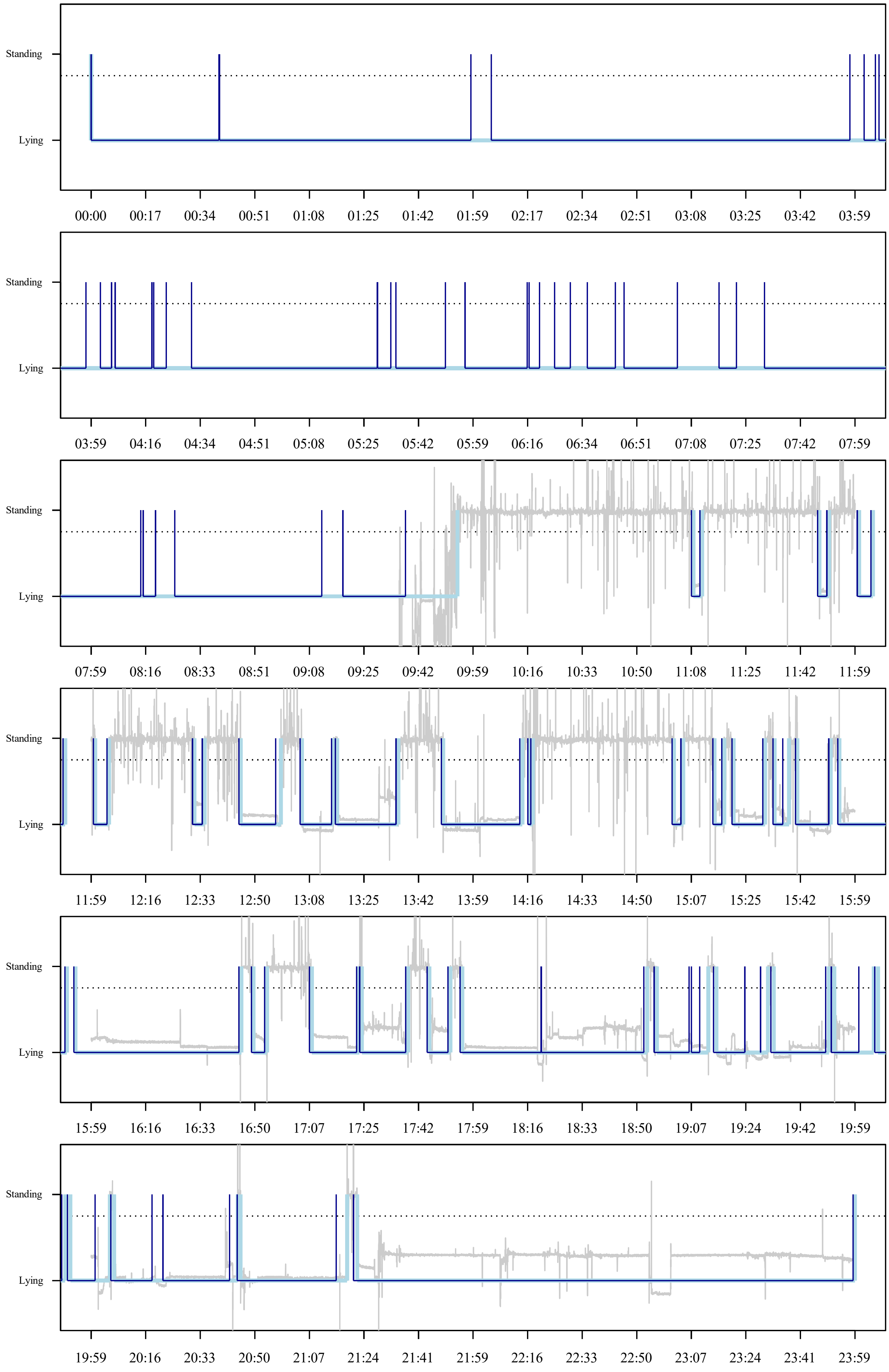

Standing
Lying
00:00
00:17
00:34
00:51
01:08
01:25
01:42
01:59
02:17
02:34
02:51
03:08
03:25
03:42
03:59
04:16
04:34
04:51
05:08
05:25
05:42
05:59
06:16
06:34
06:51
07:08
07:25
07:42
07:59
08:16
08:33
08:51
09:08
09:25
09:42
09:59
10:16
10:33
10:50
11:08
11:25
11:42
11:59
12:16
12:33
12:50
13:08
13:25
13:42
13:59
14:16
14:33
14:50
15:07
15:25
15:42
15:59
16:16
16:33
16:50
17:07
17:25
17:42
17:59
18:16
18:33
18:50
19:07
19:24
19:42
19:59
20:16
20:33
20:50
21:07
21:24
21:41
21:59
22:16
22:33
22:50
23:07
23:24
23:41
23:59

8863

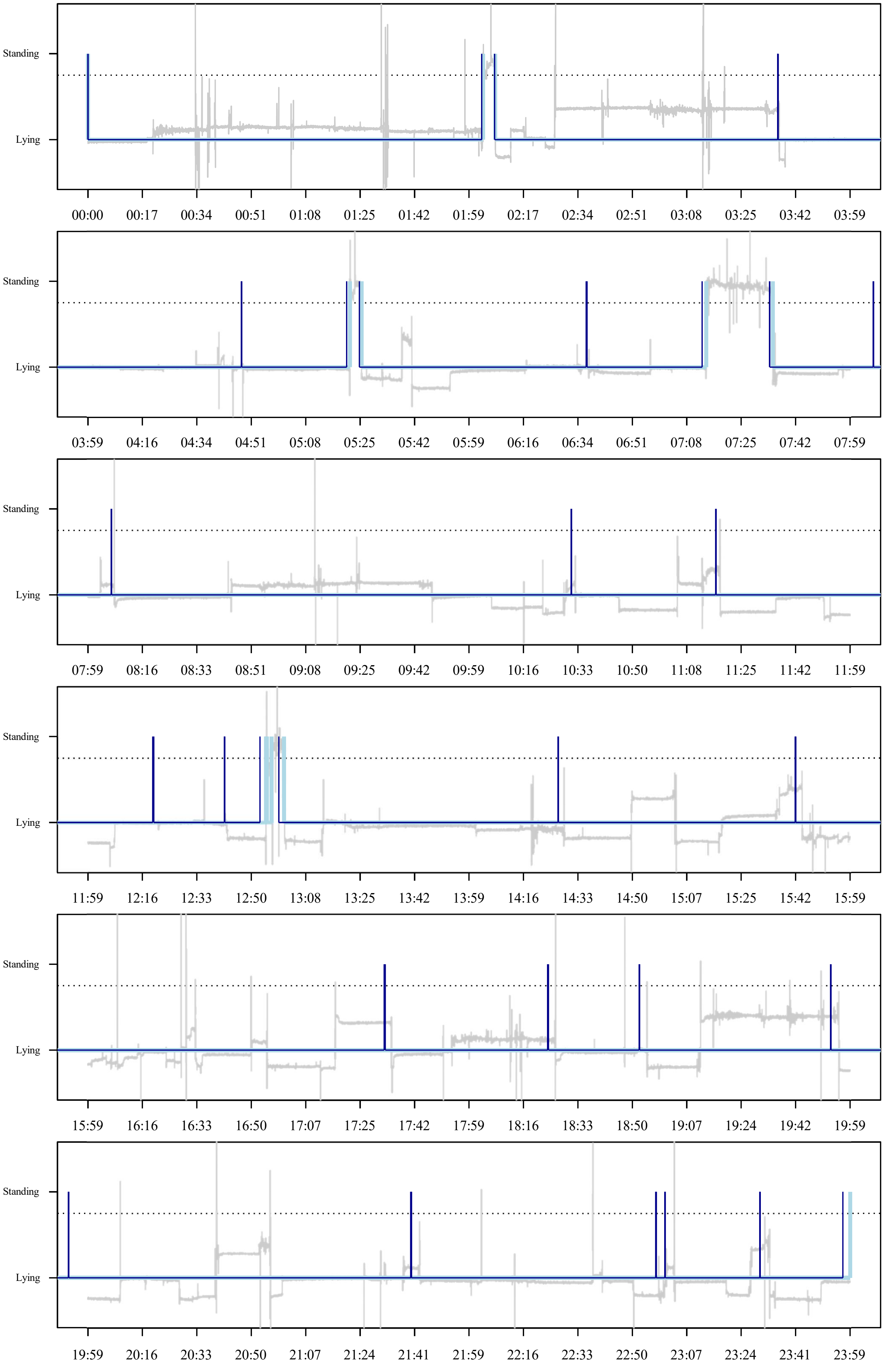
Standing
Lying
00:00 00:17 00:34 00:51 01:08 01:25 01:42 01:59 02:17 02:34 02:51 03:08 03:25 03:42 03:59
03:59 04:16 04:34 04:51 05:08 05:25 05:42 05:59 06:16 06:34 06:51 07:08 07:25 07:42 07:59
07:59 08:16 08:33 08:51 09:08 09:25 09:42 09:59 10:16 10:33 10:50 11:08 11:25 11:42 11:59
11:59 12:16 12:33 12:50 13:08 13:25 13:42 13:59 14:16 14:33 14:50 15:07 15:25 15:42 15:59
15:59 16:16 16:33 16:50 17:07 17:25 17:42 17:59 18:16 18:33 18:50 19:07 19:24 19:42 19:59
19:59 20:16 20:33 20:50 21:07 21:24 21:41 21:59 22:16 22:33 22:50 23:07 23:24 23:41 23:59
